# Tuberculosis to lung cancer: application of tuberculosis signatures in identification of lung adenocarcinoma subtypes and marker screening

**DOI:** 10.7150/jca.97898

**Published:** 2024-08-13

**Authors:** Fan Feng, Wanjie Xu, Chaoqun Lian, Luyao Wang, Ziqiang Wang, Huili Chen, Xiaojing Wang, Hongtao Wang, Jing Zhang

**Affiliations:** 1 Anhui Provincial Key Laboratory of Immunology in Chronic Diseases, Research Center of Laboratory Medicine, School of Laboratory Medicine, Bengbu Medical University, Bengbu, 233030, China.; 2 School of Biological and Food Engineering, Suzhou University, Anhui 234000, China.; 3 Department of Genetics, School of Life Sciences, Bengbu Medical University, Bengbu, 233030, China.; 4 Department of Clinical Medicine, Bengbu Medical University, Bengbu, 233030, China.; 5 Research Center of Clinical Laboratory Science, Bengbu Medical University, Bengbu, 233030, China.; 6 Anhui Province Key Laboratory of Clinical and Preclinical Research in Respiratory Disease, Molecular Diagnosis Center, Joint Research Center for Regional Diseases of IHM, First Affiliated Hospital, Bengbu Medical University, Bengbu, 233030, China.

**Keywords:** lung adenocarcinoma, pulmonary tuberculosis, molecular subtypes, immunophenotyping, prognostic model

## Abstract

**Background**: There is an association between LUAD and TB, and TB increases the risk of lung adenocarcinogenesis. However, the role of TB in the development of lung adenocarcinoma has not been clarified.

**Methods**: DEGs from TB and LUAD lung samples were obtained to identify TB-LUAD-shared DEGs. Consensus Clustering was performed on the TCGA cohort to characterize unique changes in TB transcriptome-derived lung adenocarcinoma subtypes. Prognostic models were constructed based on TB signatures to explore the characterization of subgroups. Finally, experimental validation and single-cell analysis of potential markers were performed.

**Results**: We characterized three molecular subtypes with unique clinical features, cellular infiltration, and pathway change manifestations. We constructed and validated TB-related Signature in six cohorts. TB-related Signature has characteristic alterations, and can be used as an effective predictor of immunotherapy response. Prognostically relevant novel markers *KRT80*, *C1QTNF6*, and *TRPA1* were validated by RT-qPCR. The association between *KRT80* and lung adenocarcinoma disease progression was verified in Bulk transcriptome and single-cell transcriptome.

**Conclusion**: For the first time, a comprehensive bioinformatics analysis of tuberculosis signatures was used to identify subtypes of lung adenocarcinoma. The TB-related Signature predicted prognosis and identified potential markers. This result reveals a potential pathogenic association of tuberculosis in the progression of lung adenocarcinoma.

## Introduction

Lung cancer is a heterogeneous disease with a wide range of clinicopathologic features and is a cancer with high morbidity and mortality in the world 1. Hundreds of thousands of people die of lung cancer worldwide every year 2. Of these, non-small cell lung cancer (NSCLC) is the most common cancer worldwide, accounting for approximately 55-60% of lung cancer deaths 3. As a common type of NSCLC, lung adenocarcinoma (LUAD) often leads to poor prognosis 4 due to unique cellular and molecular features 5. Patients are often in the metastatic stage at the time of diagnosis when the malignancy can no longer be treated surgically 6, 7. Therefore, it is of great value to explore reliable markers that can accurately estimate the clinical prognosis and response to treatment 8.

Tuberculosis is an infectious disease caused by Mycobacterium tuberculosis (Mtb) and is one of the top ten causes of death worldwide 9. The lung is the most commonly affected organ in tuberculosis infections 10, and the prolonged presence of Mtb in the lungs leads to a range of pathologic outcomes, such as the formation of granulomas and cavities 11 and the malignant transformation of localized tissues that undergo repetitive damage and repair 12. Tuberculosis (TB) accounts for about 85% of clinical tuberculosis cases 13, and its occurrence may be associated with a variety of lung diseases. For example, TB infection has now been shown in different studies to increase the probability of lung cancer 11, 14, especially lung adenocarcinoma 15, 16. Also, research has shown a higher mortality rate in cases of tuberculosis combined with lung cancer, not only because patients with tuberculosis may delay the diagnosis of lung cancer, which increases lung cancer morbidity and mortality 17. Although tuberculosis has been recognized as a risk factor for the development of lung cancer, previous findings remain conflicting and uncertain due to the potential confounding of smoking and other comorbidities 18, so it is critical to distinguish between the two diseases accurately. In addition to the link between the two diseases in clinical studies not being clear, similar transcriptomic alterations in the shared biological processes of the two diseases and the potential pathogenic links have likewise not been articulated. Considering the potential association between the two diseases and the existence of delayed diagnosis, a comprehensive exploration of the potential associations and pathogenic links between TB and LUAD at the bioinformatics level demonstrates significant value.

In this study, we discovered TB-LUAD-shared DEGs based on lung transcriptome data and screened for key LUAD pathogenic mediators affected by TB infection, i.e., TB Signature genes. Molecular subtypes characterized with Immune inflamed, Immune exclued, and Immune desert were identified by Consensus Clustering, and the unique manifestations of the subtypes in clinical features, Cellular infiltration, and pathway change revealed distinctive alterations in the transcriptome-derived lung adenocarcinoma subtypes of tuberculosis. The TB-related Riskscore was constructed based on The Least absolute shrinkage and selection operator (LASSO) Cox regression analysis and its association with Clinical characteristics, Cellular infiltration, Mutations atlas, and immunotherapy efficacy, and found that Riskscore could accurately predict the prognosis and response to immunotherapy in lung adenocarcinoma patients. Finally, we verified the expression and potential roles of potential markers by RT-qPCR experiments and single-cell analysis. We aimed to show that tuberculosis and lung adenocarcinoma have potentially shared biological processes and precise pathogenic links, providing new ideas and insights for studying characteristic markers for LUAD diagnosis and treatment.

## Materials and Methods

### Data collection and pre-processing

Lung adenocarcinoma sample sequencing data and clinical characterization were collected through publicly available datasets from the NCBI GEO (https://www.ncbi.nlm.nih.gov/geo/), TCGA (https://cancergenome.nih.gov/) databases. The TCGA cohort, GSE50081 cohort 19, GSE31210 cohort 20, GSE37745 cohort 21, GSE72094 cohort 22, GSE30219 cohort 23, IMvigor210 cohort 24, GSE78220 cohort 25 and GSE148036 cohort 26 data were processed separately. LUAD RNA sequencing data from the TCGA cohort were downloaded in The Cancer Genome Atlas (TCGA) via The Genomic Data Commons (https://portal.gdc.cancer.gov/), and subsequent analyses were performed using the TPM format for RNA sequencing data. Sample inclusion criteria for TCGA were samples containing complete survival information and expression profiles, and exclusion of samples with a survival time of less than 20 days. RNA sequencing data of lung adenocarcinoma samples, tuberculosis samples, and immunotherapy samples were collected from the GEO databases GSE50081, GSE31210, GSE37745, GSE72094, GSE30219, GSE148036 (Supplementary Table 1), and GSE78220. among them, GSE50081, GSE37745, GSE30219 were extracted from which lung adenocarcinoma samples were extracted for subsequent analysis.

### Differential analysis and screening of signature genes

To identify Differentially Expressed Genes (DEGs) in diseases, the DESeq2 package (v1.36.0) was utilized to assess differentially expressed genes between subgroups. The significance screening criteria for related genes were adj.P.Value < 0.05, |logFC| > 1.5. Differential genes in TB and lung adenocarcinoma were compared, and intersecting genes, defined as TB-LUAD-shared DEGs, were obtained. Kyoto Encyclopedia of Genes and Genomes (KEGG) enrichment analysis of TB-LUAD-shared DEGs was performed using clusterProfiler R package (v4.6.2). Genes associated with overall survival (OS) (P<0.05) were further screened in the TCGA cohort using Univariate Cox regression analysis and Kaplan-Meier analysis and defined as TB Signature genes.

### Consensus clustering to identify subtypes

Consensus Clustering was performed using ConsensusClusterPlus (v1.62.0). The optimal number of clusters was assessed by Cumulative Distribution Function (CDF) plots and Consensus Heatmaps, with an optimal K-value of 3. Kaplan Meier analyses were used to assess the differences in Overall Survival (OS) between clusters. Univariate Cox regression analysis and Multivariate Cox regression analysis were performed using survival (v3.5-7) and survminer (v0.5.6) to assess characteristics as independent predictors of survival. TB signature gene expression heatmaps were performed using the pheatmap (v1.0.12) package. DEGs for molecular subtypes were obtained by comparison of individual subtypes with the remaining subtypes.

### Differences in molecular pathways between subtypes

Well-defined biosignatures were obtained from the human Hallmark gene set (h.all.v2023.1.Hs.symbols). Accessed via the Molecular Signatures Database (MSigDB, http://software.broadinstitute.org/gsea/msigdb/). The GSVA (v1.44.5) package was utilized to study changes in biological processes between clusters. PROGENy enrichment was performed using the progeny (v1.18.0) package to quantify signaling pathway target gene enrichment 27 to clarify pathway alterations between subgroups further. The Mariathasan gene set was curated by Mariathasan S et al. 24, and GSVA was used to quantify the extent of pathway changes in the samples.

### Tumor microenvironment (TME) infiltration exploration

The ESTIMATE (v1.0.13), IOBR (v0.99.9) R package 28 was utilized to perform the ESTIMATE algorithm and Immuno-Oncology Biological Research (IOBR) analyses to investigate tumor microenvironmental characteristics of each LUAD sample. The IOBR package integrates eight published algorithms for quantifying tumor microenvironment (TME) algorithms: CIBERSORT, TIMER, xCell, MCPcounter, ESITMATE, EPIC, IPS, quanTIseq, which allows for a more comprehensive analysis of cellular infiltration levels in the TME. The cancer-immunity cycle was derived from Xu et al. 29 and was analyzed by expression scores obtained by expression profiling and compared between groups. Twenty-eight immune cell gene sets were collected from Charoentong P et al 30.

### Somatic mutation analysis

Somatic mutation and CNV data for the TCGA cohort were downloaded from GDC TCGA (https://cancergenome.nih.gov/). After acquiring the data, the maftools (v2.12.0) R package was utilized to explore the variability of gene mutations between subgroups.

### Construction and validation of TB-related prognostic models

The Least absolute shrinkage and selection operator (LASSO) COX regression analysis was performed using the R packages glmnet (v4.1-8), survival (v3.5-7), and survminer (v0.4.9), which were used to construct a tuberculosis characterization-based lung adenocarcinoma risk model and derived the riskscore equation Riskscore = Ʃ (βi × Expi). The βi coefficient represents the weight of the respective marker, and Expi represents the expression value. The prognostic value of the score was validated in the TCGA cohort, GSE50081 cohort, GSE31210 cohort, GSE37745 cohort, GSE72094 cohort, and GSE30219 cohort.

For the genes in the prognostic model, Kaplan-Meier analysis was performed using the survival (v3.5-7) package to assess the relationship with overall survival (OS) in terms of median high and low mRNA expression groups. The diagnostic value of the genes was evaluated using the Receiver Operating Characteristic Curve (ROC) analysis utilizing the pROC (v1.18.4) R package.

### The predictive efficacy of TB-related Riskscore in Immunotherapy Response

The IMvigor210 cohort and GSE78220 cohort were utilized to validate the predictive value of TB-related Riskscore in immunotherapy response. In addition, specific immunotherapy datasets were used to obtain immunotherapy data 31. Using the TCGA cohort as the low-risk and high-risk group data, the GenePattern website's Submap model was used to predict the differences in immunotherapy between risk groups to determine the efficacy of anti-PD-1 and anti-CTAL4 in patients with different scores and to improve the accuracy of the clinical prognostic predictors.

### Screening and validation of potential markers

Model genes were analyzed using The Gene Set Cancer Analysis (GSCA) database 32. The relationship between KRT80 expression and tumor stage was visualized using the ggpubr (v0.6.0) package. The expression of *KRT80* protein in normal and lung adenocarcinoma samples was analyzed in The University of ALabama at Birmingham CANcer data analysis Portal (UALCAN) database 33 and the relationship between its expression and alterations in the WNT pathway was explored. TCGA samples were categorized into high and low expression groups according to the median KRT80 expression, and the Gene set enrichment analysis (GSEA) was performed using limma (v3.54.0), clusterProfiler (v4.6.2) R packages, to explore the relationship between *KRT80* expression and Hallmark pathway relationships. Reactome pathway enrichment analysis was performed on the high- and low-expression groups using the ReactomePA (v1.40.0) package to explore more detailed pathway associations.

Single-cell RNA sequencing data from 8 LUAD patients were obtained from Bischoff et al 34. Data were transformed into Seurat objects using the Seurat package (v4.4.0) 35. Quality control of the data was first performed, specifically retaining genes expressed in at least three single cells, removing cells with less than 200 or more than 4000 expressed genes, and retaining cells with less than 15% mitochondrial genes. The NormalizeData function was used to normalize the data. Clustering was performed using FindClusters and FindNeighbors, and the results were visualized using UMAP and annotated using SingleR (v2.2.0) 36. Cell trajectories were inferred using the Monocle2 37 algorithm for the gene-cell matrices extracted from the Seurat subset as input data.

### Cell culture and RT-qPCR

All cells were cultured in an incubator at 37 °C in a 5% CO2 atmosphere. Human normal lung bronchial epithelial cell line BEAS-2B, lung adenocarcinoma cell line A549, and NCI-H1299 were from the Chinese Academy of Sciences (Shanghai, China). Cell culture media, culture dishes, and petri dishes were obtained from Thermo Fisher Scientific (Invitrogen, USA) and Corning Incorporated. RNA was extracted from cell lines as a control. SYBR Green qPCR mix (Vazyme, China) was used to synthesize cDNA for real-time PCR. Our results were analyzed using the comparative Ct method, and the Ct value of each gene was normalized by the Ct read of the corresponding GAPDH. All data are expressed as mean ± standard deviation (SD) of three independent experiments. Primer sequences are shown in the Supplementary Table (Supplementary Table 2).

### Statistical analyses

Statistical analysis and academic graphing were performed in R software (v3.6.3) and GraphPad Prism 8.0. Two-by-two comparisons between the two groups were performed using the Wilcoxon test and t-test, and survival analysis was performed using the Kaplan-Meier method and log-rank test. The statistical significance of cell line experiments was assessed using GraphPad Prism version 9 software. Differences were considered statistically significant at *p < 0.05, **p < 0.01, and ***p < 0.001.

## Results

### Characterizing TB signature genes to identify the molecular subtypes

**Figure 1:**
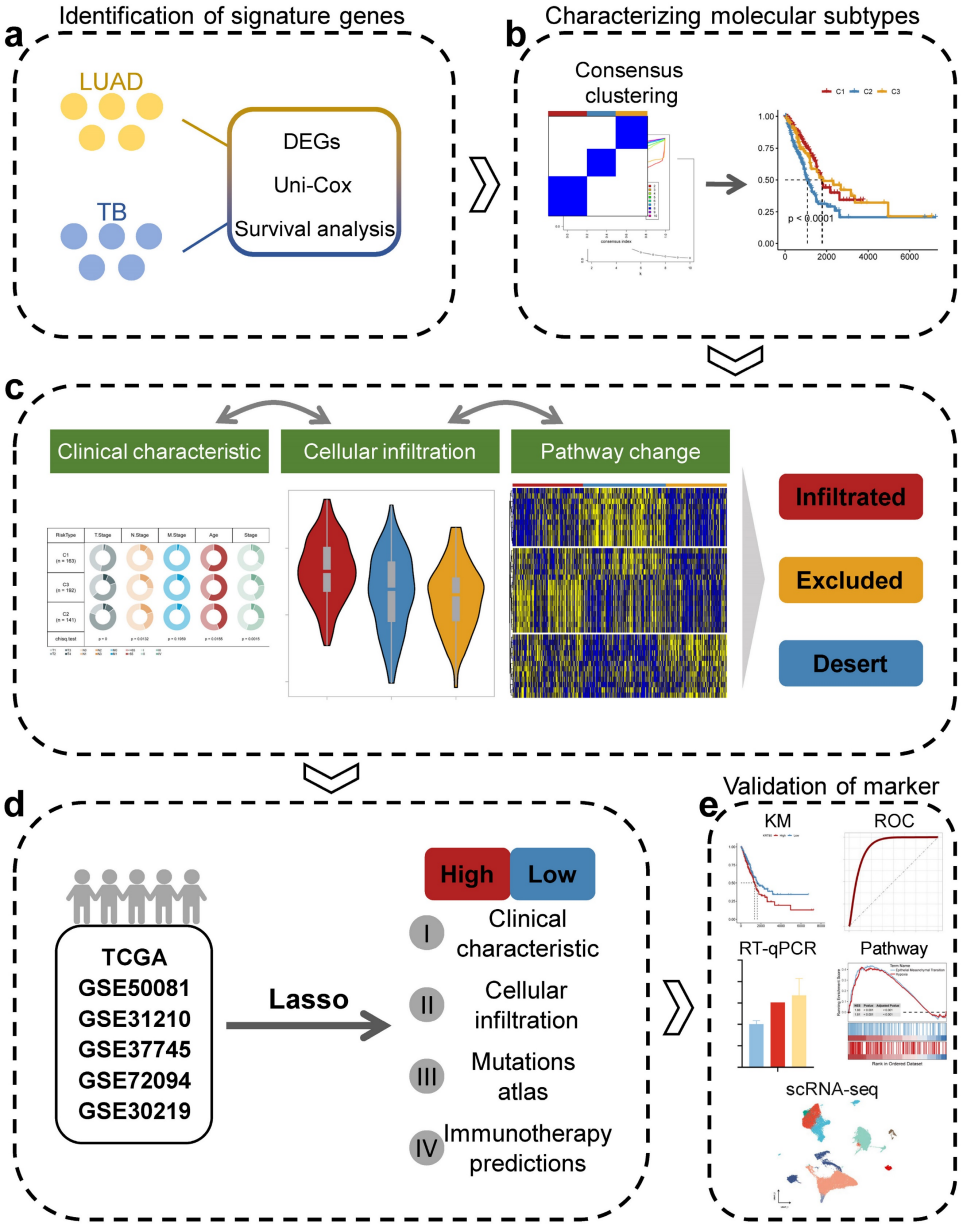
The overall workflow of this study.

**Figure 2:**
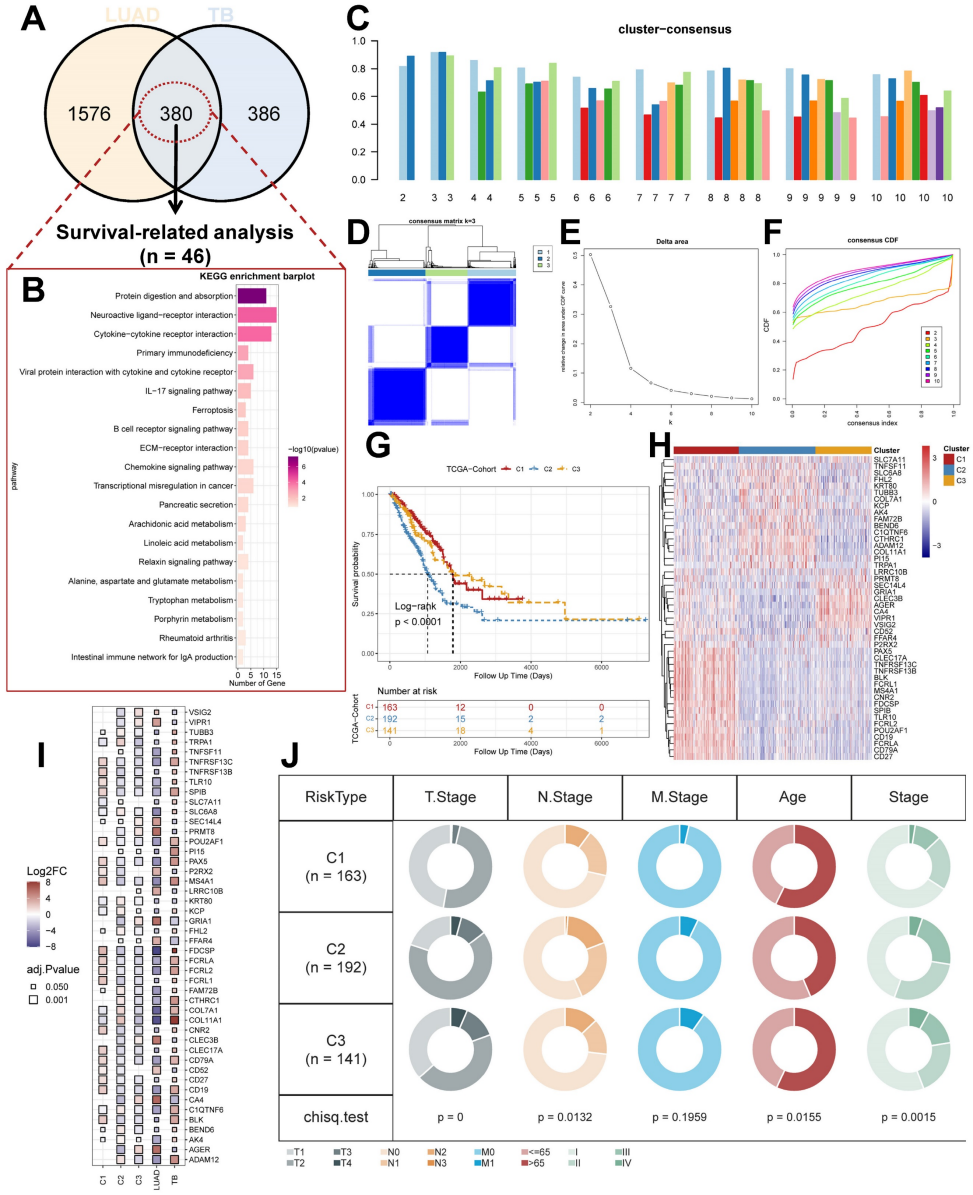
** Identification of unique LUAD molecular subtypes derived from TB transcriptional profiles.** (A) Distribution of DEGs for TB and LUAD. (B) KEGG enrichment analysis results for TB-LUAD-shared DEGs. (C) The cluster-consensus results for the TCGA cohort. (D) Heatmap depicting consensus clustering solution (k = 3) for TB Signature genes in TCGA cohort. (E) Delta area curve of Consensus Clustering. (F) Empirical cumulative distribution function (CDF) plots display consensus distributions for each k. (G) The Kaplan-Meier curves for C1, C2, and C3 subtypes in the TCGA cohort. (H) Heatmap depicting the expression of genes in three subtypes. (I) Differential expression of TB Signature genes in subtypes. (J) Distribution of clinical features of subtypes.

Prior to the commencement of the entire study, we summarized the study design ideas and overall workflow to provide a general overview of the way in which molecular subtypes of lung adenocarcinoma were derived, and potential markers of lung adenocarcinoma were explored based on TB characteristics (Figure 1). Firstly, we processed the data of GSE148036 and compared the TB and LUAD samples with normal samples for the DESeq2 package for differential analysis of count data. We screened the genes that were differentially expressed in TB and LUAD and obtained the 380 common genes in the differential genes of TB and LUAD. This represents a common alteration in the lung transcriptional profile between TB and LUAD and was therefore defined as TB-LUAD-shared DEGs (Figure 2A and Supplementary Table 3). We performed KEGG enrichment analysis to explore the biological processes of TB-LUAD-shared DEGs. The results showed that these genes were mainly associated with extracellular matrix (ECM) pathways, immune responses, cell growth and proliferation, and metabolic processes. In particular, tryptophan metabolism has a complex impact on immune escape in lung cancer 38; the IL-17 signaling pathway is important for intracellular bacterial clearance 39, 40 and is regulated during Mycobacterium tuberculosis infection 41-43. This result suggests that TB-LUAD-shard DEGs may be key LUAD-related pathogenic mediators affected by TB infection (Figure 2B).

We performed Univariate Cox proportional hazards regression analysis (P < 0.05) and Kaplan-Meier analysis (P < 0.05) on TB-LUAD-shared DEGs. We finally obtained 46 genes associated with OS in LUAD patients and defined them as TB Signature genes. Using 46 TB Signature genes as input genes, the TCGA cohort was identified into three molecular subtypes by Consensus Clustering, i.e., Cluster1 (C1), Cluster2 (C2), and Cluster3 (C3) (Figure 2C-2F). The results of the Kaplan-Meier analysis showed significant prognostic differences among the three subtypes. The C1 subtype had the best prognosis, followed by the C3 subtype, and the C2 subtype had the worst prognostic profile (P < 0.0001, log-rank test, Figure 2G). There was a significant difference in the expression of the TB signature genes between the subtypes (Figure 2H and 2I), suggesting a differential prognostic outcome for patients with LUAD under the influence of the transcriptome signature of TB. Univariate Cox regression analysis showed that subtype could be an independent prognostic factor, in which C2 was associated with poor prognosis (C1 vs. C2, HR 2.02 [95% CI, 1.408 - 2.90], P < 0.001; Supplementary Figure 1A). Multivariate Cox regression analysis also showed that molecular subtypes were associated with survival outcomes in LUAD patients after adjustment for clinical case factors, with C2 associated with poor prognosis (C1 vs. C2, HR 1.740 [95% CI, 1.193 - 2.54], P=0.004; Supplementary Figure 1B). In addition, we summarized the clinical features of the three subtypes (Figure 2J). The results showed that C2 and C3 subtypes existed with a greater proportion of the occurrence of T4, M1, and IV stage progression, suggesting the possibility of higher tumor metastasis and malignant progression.

### Molecular processes and immunological characterization of subtypes

**Figure 3:**
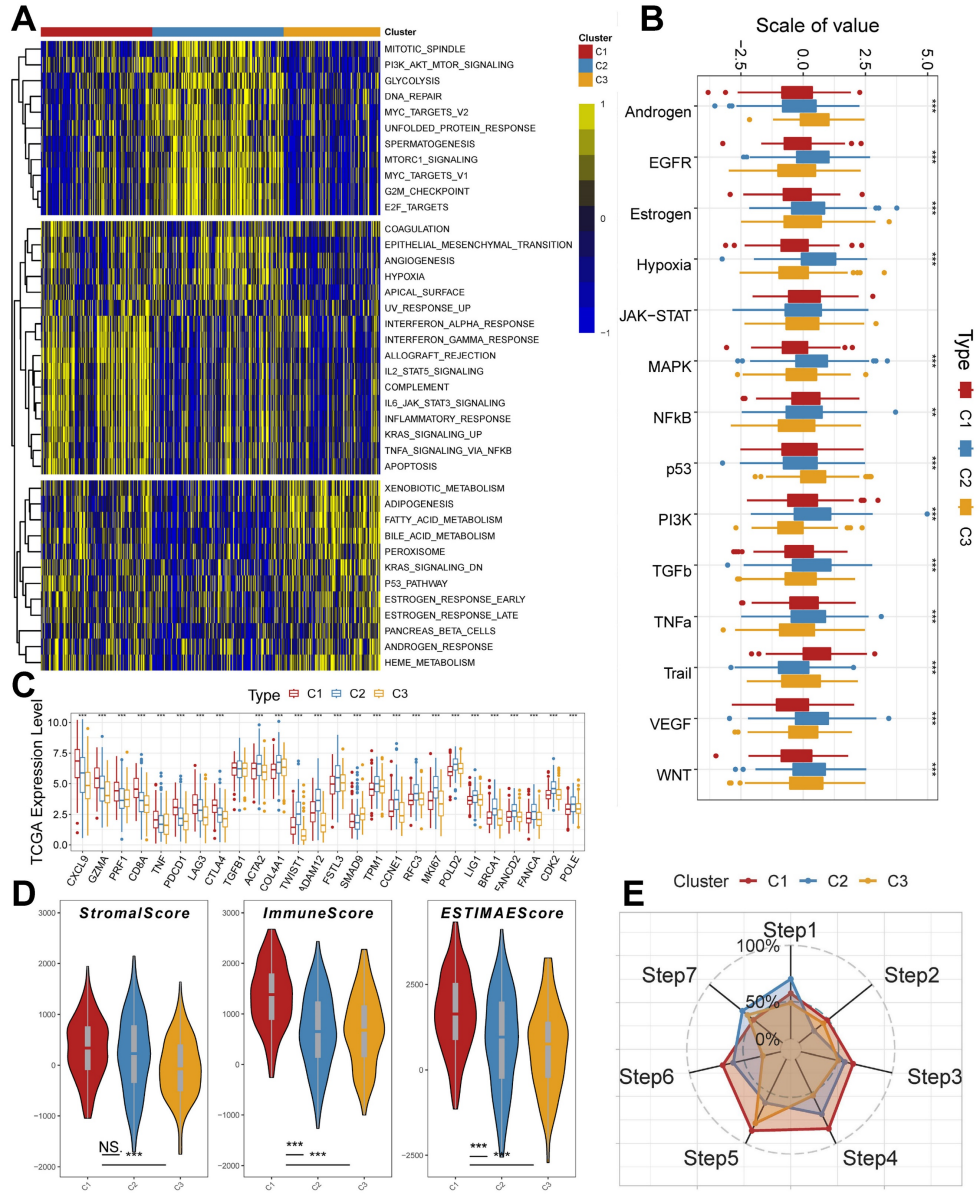
** Unique molecular processes and immunological features of molecular subtypes.** (A) Heatmap depicting the distribution of subtypes in the Hallmark signaling set. (B) PROGENy probing the altered tumor signaling pathways of molecular subtypes (Kruskal test). (C) Expression of transcriptional markers among subtypes (Kruskal test). (D) ESTIMATE scores for molecular subtypes (Kruskal test). (E) Anti-cancer immunoreactivity of the subtypes in the cancer-immunity cycle. *, p < 0.05. **, p < 0.01. ***, p < 0.001.

To further explain the variability of molecular subtypes in clinical features, we explored the biological molecular alterations between subtypes. We performed gene set variation analysis (GSVA) using the Hallmark gene set (Figure 3A), and the GSVA results showed that C1 was significantly enriched in immune infiltration-related pathways, including INF-α response, INF-γ response, allograft rejection, IL2 STAT5 signaling, IL6 JAK STAT3 signaling, and inflammatory response, etc.; the enriched pathways of C2 were associated with PI3K AKT mTOR signaling, glycolysis, DNA repair, MYC targets V1/V2, G2M checkpoint, E2F targets, mitotic spindle, Unfolded Protein Response, etc. with significant oncogenic activation and high proliferation characteristics; C3 was highly enriched in stroma-associated signaling pathways, including adipogenesis, P53 pathway, etc. (Supplementary Figure 1C). Interestingly, partial immune activation and immune cell infiltration were likewise observed in C3. This is consistent with the previously reported immune rejection phenotype in which immune cells are retained in the peripheral stroma of tumor cells rather than penetrating their stroma 44, 45. Meanwhile, Unfolded Protein Response (UPR), which is highly expressed in C2, as one of the most important adaptive systems of tumor cells, can adapt to external stimuli by integrating multiple signaling pathways to promote tumor cell survival, which has been shown to be associated with EMT 46. PROGENy enrichment analysis further validated the molecular pathway differences between subgroups (Figure 3B), which showed that C2 and C3 were significantly enhanced in pathways related to tumor development and metastasis, including EGFR, Hypoxia, MAPK, PI3K, VEGF, and WNT pathways. We also analyzed the expression of inflammatory, stromal-related mRNAs in the subtypes to explore the relationship between the three clusters and the molecular perturbation environment (Figure 3C). *CXCL9*, *GZMA*, *PRF1*, *CD8A*, *TNF*, *PDCD1*, *LAG3*, and *CTLA4* were considered immune activation-related transcripts; *TGFB1*, *ACTA2*, *COL4A1*, *TWIST1*, *ADAM12*, *FSTL3*, *SMAD9*, and *TPM1* were considered TGF-β/EMT pathway-related transcripts; and *CCNE1*, *RFC3*, *MKI67*, *POLD2*, *LIG1*, *BRCA1*, *FANCA*, *FANCD2*, *CDK2*, and *POLE* were considered cell cycle/proliferation-related transcripts. We found that mRNA associated with immune activation pathways were significantly upregulated in C1, suggesting that this subtype is considered as an immune activation group. In contrast, mRNAs associated with stromal activation and cell proliferation-related transcripts were highly expressed in C2 and C3. In addition, we performed enrichment analysis using the gene set curated by Mariathasan et al. The results showed significantly elevated immune activation in C1, significant activation of oncogenic pathways in C2, and significantly enhanced stromal and angiogenic activity in C3, which confirmed our speculations (Supplementary Figure 2A).

The tumor microenvironment (TME) occupies an important role in promoting lung carcinogenesis 47, and we analyzed the cellular infiltration of molecular subtypes. We first quantified the overall immune infiltration level of molecular subtypes using the ESTIMATE algorithm (Figure 3D). We found that ImmuneScore and StromalScore had a higher level in C1, which suggests that we have more immune cell infiltration in C1 compared to C2 and C3. Systematic tracking of tumor immunophenotypes is essential for understanding tumor immune mechanisms and improving the clinical efficacy of immunotherapy. To further explore the unique immune progression of tuberculosis-derived LUAD subtypes, we analyzed the cancer-immunity cycle among subtypes (Figure 3E). The results showed that the level of C2 was significantly enhanced in the first step: Release of cancer cell antigens, and the seventh step: Killing of cancer cells; while C1 was significantly enhanced in the second step: Cancer antigen presentation, and the third step: Priming and activation, Step 4: Trafficking of immune cells to tumors, Step 5: Infiltration of immune cells into tumors, and Step 6: Recognition of cancer cells by T cells were all expressed at higher levels. This correlates with the presence of more immune cell infiltration and stronger immune activation in C2.

### Unique cellular infiltration landscapes and genomic alterations in subtypes

**Figure 4:**
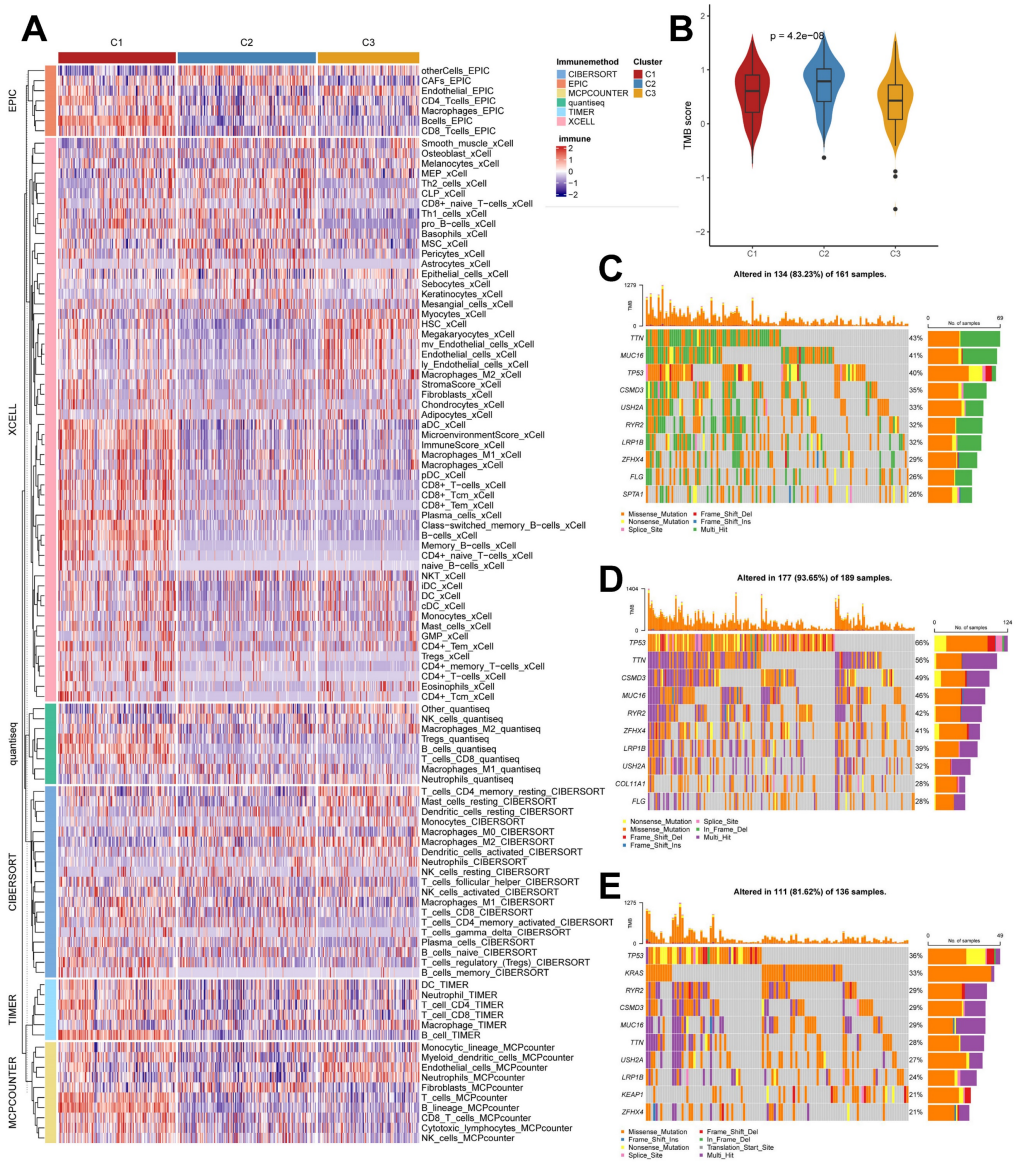
** Cellular infiltration landscapes and mutations of subtypes.** (A) Heatmap demonstrating the variability in cellular infiltration levels between subtypes. (B) TMB scores of subtypes (Kruskal test). (C-E) Unique mutational landscapes of the three subtypes.

We used the IOBR algorithm to explore the variability of cellular infiltration patterns in the subtypes and found that most cell classes differed significantly among the three subtypes (Figure 4A). C1 was dominated by CD4+ T cells, CD8+ T cells, B cells, and macrophages M1; C2 was characterized by Smooth muscle cells, Th2 cells, CAFs, Basophils, and other cells with upregulated infiltration; C3 was enriched in endothelial, neutrophils, epithelial cells, monocytes, and myocytes. We also obtained similar results in the ssGSEA algorithm (Supplementary Figure 2B), with more infiltration of activated B cells, activated CD4 T cells, activated CD8 T cells, immature B cells, and macrophages present in the C2 subtype. We also analyzed their somatic mutation patterns. The results showed that the tumor mutation burden (TMB) was significantly elevated in C2 (Figure 4B). This result is consistent with the cancer-immunity cycle, i.e., high TMB subtypes have the potential for more tumor antigen production, which in turn promotes step one and step seven. We also noted significant mutational differences in the subtypes in the first ten genes (Figure 4C-4E).

Previously, it has been reported in the literature that tumors can be classified into three categories based on their immunophenotypes: Immune inflamed, Immune exclued, and Immune desert. Based on the above findings in clinical features, molecular processes, and immunological characteristics, we hypothesized that the three molecular subtypes of LUAD derived from the TB transcriptome could be distinguished into three phenotypes with distinctive features (Supplementary Table 4). The C1 subtype is Immune inflamed, characterized by INF-γ signaling expression, B-cell infiltration, as well as massive immune cell infiltration and inflammatory response; the C2 subtype is Immune desert, characterized by tumor cell proliferation, glycolysis, WNT Signaling expression, and immunosuppression; and the C3 subtype is Immune excluded, characterized by stromal activation, angiogenesis, and immune cell infiltration and exclusion. This result also indicates that the progression of LUAD under the effect of TB causative factors can have diverse manifestations and different prognoses.

### Construction and validation of TB-related Riskscore

**Figure 5:**
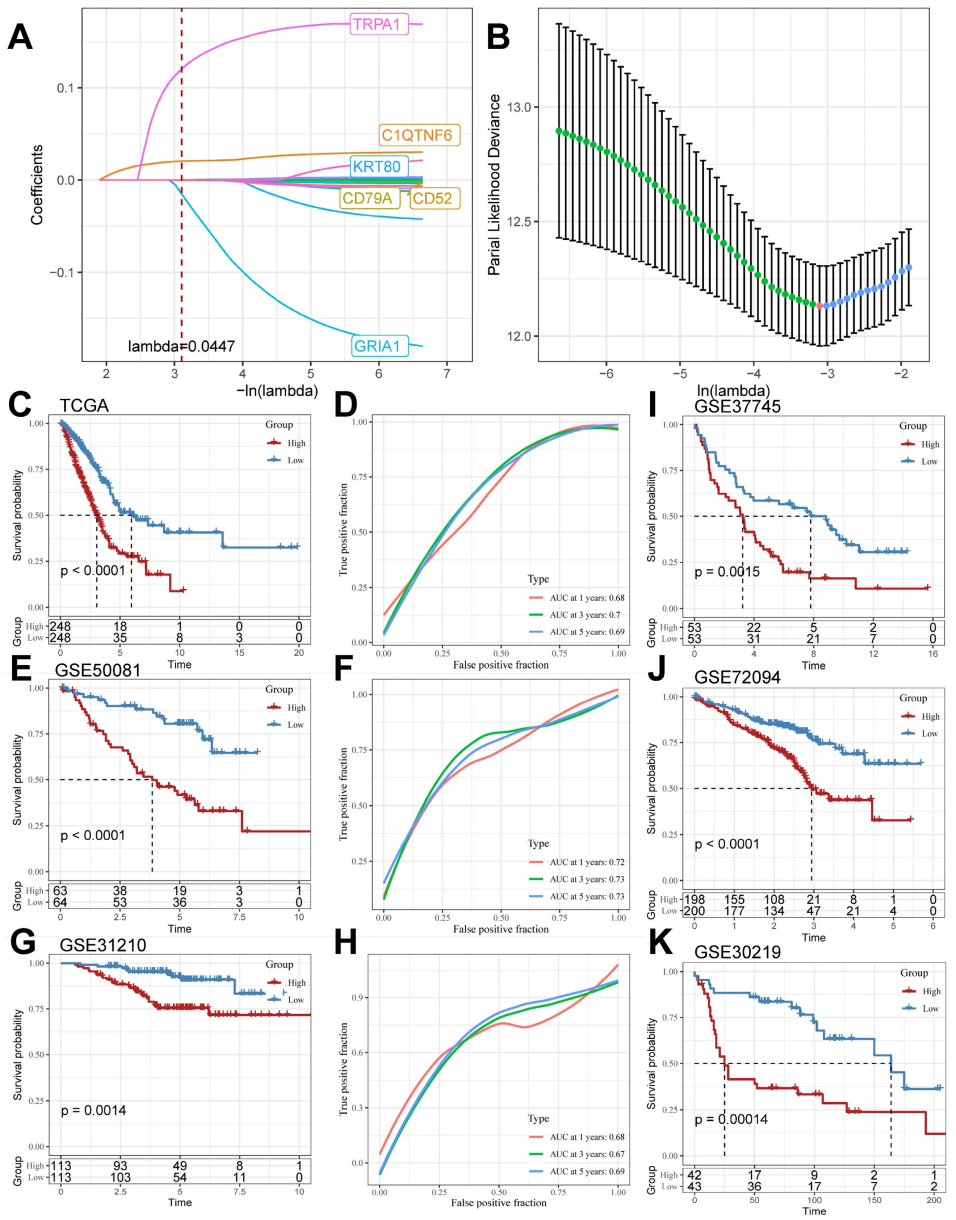
** LASSO Algorithm to construct TB-related Riskscore in multiple cohorts.** (A and B) LASSO algorithm to derive 6 model genes. (C and D) Kaplan-Meier curves for the high and low-risk score groups and the Receiver Operating Characteristic Curve analysis in the TCGA cohorts. (E and F) Kaplan-Meier curves for the high and low-risk score groups and the Receiver Operating Characteristic Curve analysis in the GSE50081 cohorts. (G and H) Kaplan-Meier curves for the high and low-risk score groups and the Receiver Operating Characteristic Curve analysis in the GSE31210 cohorts. (I-K) Kaplan-Meier curves for the high and low-risk score groups in GSE37745, GSE72094, and GSE30219 cohort.

We screened the differentially expressed genes in the TCGA cohort (logFC > |2|, adj. P.value < 0.05), compared the DEGs in the TCGA cohort with the TB Signature genes and obtained the intersections, and further mined out 19 genes in which the TB Signature genes had significant expression in LUAD. We used the 19 genes as input genes to construct a TB-related Riskscore by using the Least absolute shrinkage and selection operator (LASSO) COX regression analysis for the TCGA cohort: Riskscore = (-0.001)*exp(*CD52*) + (- 0.001)*exp(*CD79A*)+(0.022)*exp(*C1QTNF6*)+(0.001)*exp(*KRT80*)+(-0.196)*exp(*GRIA1*)+(0.172)*exp(*TRPA1*) (Figure 5A and 5B). The score of each sample was calculated using this formula, and the samples in the cohort were divided into high-risk and low-risk groups based on the median score (Supplementary Table 5). Kaplan-Meier analyses were performed to explore the difference in prognosis between the high- and low-risk groups. The Receiver Operating Characteristic Curve (ROC) is used to assess the predictive performance of the model. The results showed that the prognosis of the high-risk group in the TCGA cohort was significantly worse than that of the low-risk group (log-rank test, P < 0.0001; Figure 5C) and had good predictive value (AUC = 0.68, 0.7, 0.69; Figure 5D). Meanwhile, the scoring model was found to have good predictive value in GSE50081 (log-rank test, P < 0.0001; Figure 5E) (AUC = 0.72, 0.73, 0.73; Figure 5F), GSE31210 (log-rank test, P = 0.0014; Figure 5G) (AUC = 0.68, 0.67, 0.69; Figure 5H), GSE37745 (log-rank test, P=0.0015; Figure 5I), GSE72094 (log-rank test, P<0.0001; Figure 5J), and GSE30219 (log-rank test, P=0.00055; Figure 5K) all had significant prognostic results and good predictive value.

The nomogram showed good predictive value for T Stage, N Stage, and RiskScore for the TCGA cohort samples (Supplementary Figure 3A), and the Calibration curve showed good predictive value of the model at 1, 3, and 5 years (Supplementary Figure 3B). In addition, the RiskScore also showed a strong predictive value compared to clinical characteristics (Supplementary Figure 3C). Based on these results, we speculated that TB-related Riskscore is a good predictor and is associated with poor prognosis.

### Unique clinical characteristics of the riskscore groups

**Figure 6:**
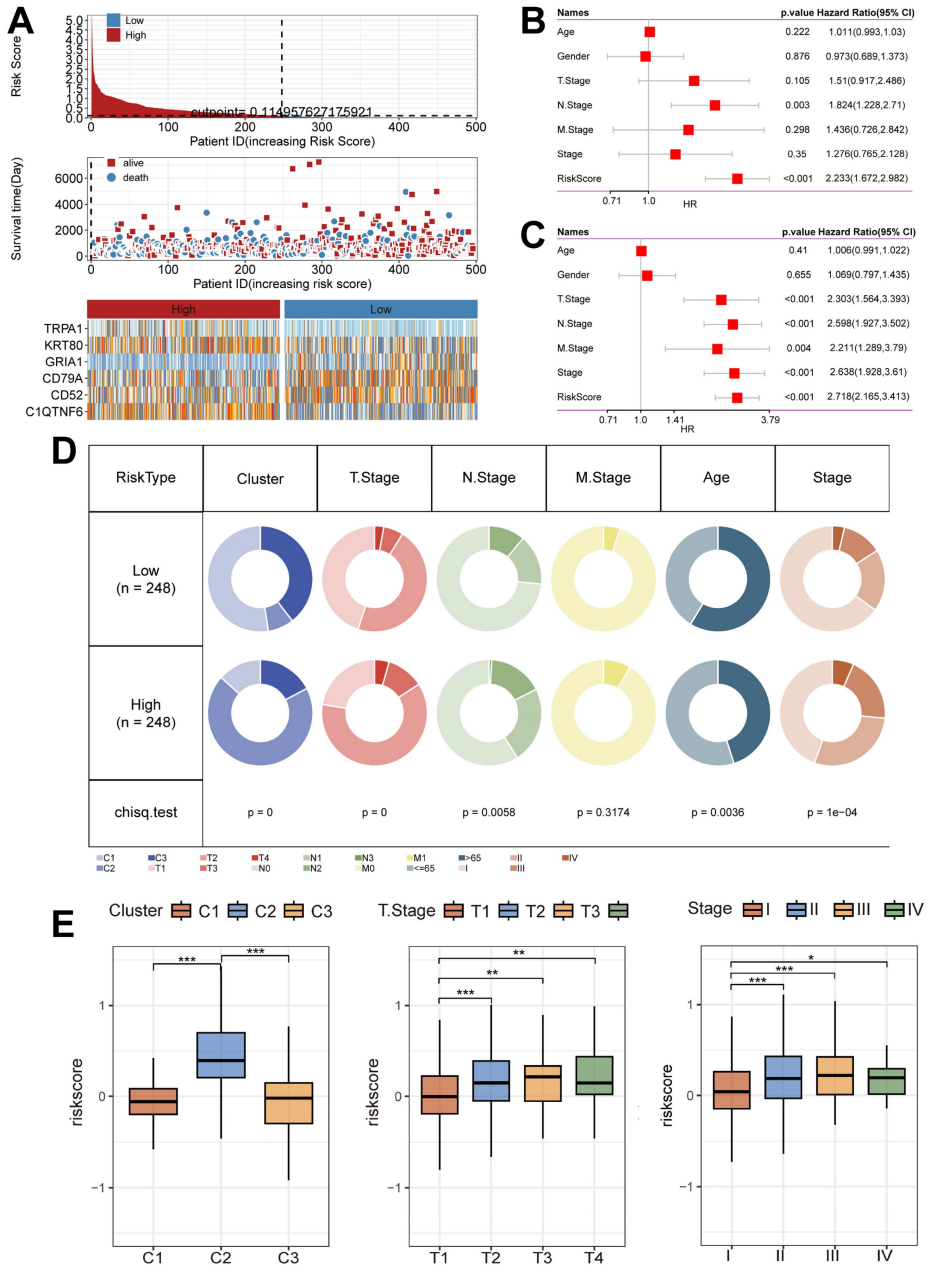
** Unique clinical characteristics of the riskscore group.** (A) Heatmap of the Riskscore distribution, patient survival, and model gene expression for the TCGA cohorts. (B) The Univariate Cox regression analysis of subtype clinical characteristics and subtyping with respect to overall survival. (C) The multivariate Cox regression analysis of subtype clinical characteristics and subtyping with respect to overall survival. (D) Distribution of clinical characteristics in high and low-risk groups. (E) Association of Riskscore with specific clinical characteristics (Wilcoxon test). *, p < 0.05. **, p < 0.01. ***, p < 0.001.

Next, we summarized the relationship between Riskscore and survival status in the TCGA cohort (Figure 6A), and the results showed that the low-risk group had a better prognostic outcome for survival compared to the high-risk group; at the same time, there was a significant difference in the modeled gene expression between the risk groups. The results of the Univariate Cox regression analysis (Figure 6B) and the Multivariate Cox regression analysis (Figure 6C) results showed that Riskscore was associated with survival outcomes and was present as a risk factor. In addition, the clinical characteristics had significantly different distributions in the high-risk and low-risk groups (Figure 6D). Notably, the C2 subtype was accompanied by high Riskscore expression, and Riskscore was statistically significant with T stage and Stage, i.e., increased Riskscore was often accompanied by the occurrence of malignant tumor progression (Figure 6E).

### TME infiltration patterns in the riskscore group

**Figure 7:**
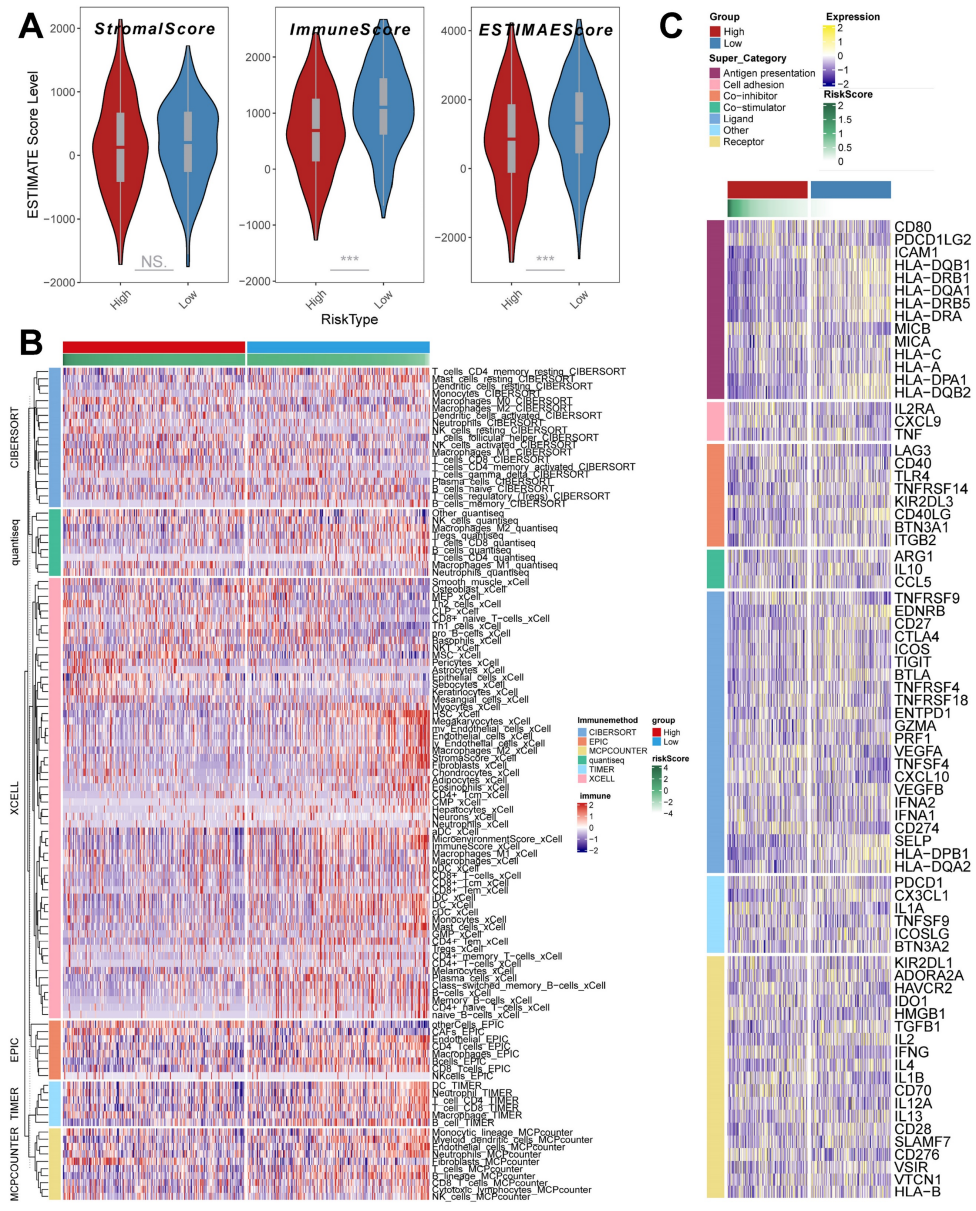
** TME infiltration pattern in the riskscore group.** (A) ESTIMATE score of riskscore groups (Wilcoxon test). (B) Heatmap demonstrating the variability in the level of cellular infiltration between riskscore groups. (C) Heatmap demonstrating the expression of immune-related transcripts between riskscore groups. *, p < 0.05. **, p < 0.01. ***, p < 0.001.

The ESTIMATE algorithm was used to quantify the overall level of immune infiltration in the two riskscore groups (Figure 7A), and we found that ImmuneScore, ESTIMATEScore was highly expressed in the low riskscore group, which suggests that there is a higher level of immune cell infiltration in the tumor microenvironment of our low riskscore group. On this basis, we utilized the IOBR algorithm to explore the variability of cellular infiltration patterns in the subgroups. We found that most cellular categories significantly differed in the two subgroups (Figure 7B). Overall, there was a decrease in immune cell infiltration and an increase in stromal cell infiltration as the score increased. And we got similar results in ssGSEA (Supplementary Figure 4A); the low-risk score group had higher levels of immune-activated cell infiltration, such as activated B cells.

In addition, we obtained transcripts associated with antigen presentation, cell adhesion, co-inhibitor, bo-stimulator, ligand, receptor, and other types 48 (Figure 7 C) and explored them in riskscore groups. The results showed significant differences in transcript expression between subgroups. In particular, we found that antigen presentation-related transcript expression was elevated as the score decreased. This result suggests that more antigen presentation processes may be occurring in the low riskscore group.

### Mutational characterization of the riskscore groups and the value in the prediction of immunotherapeutic response

**Figure 8:**
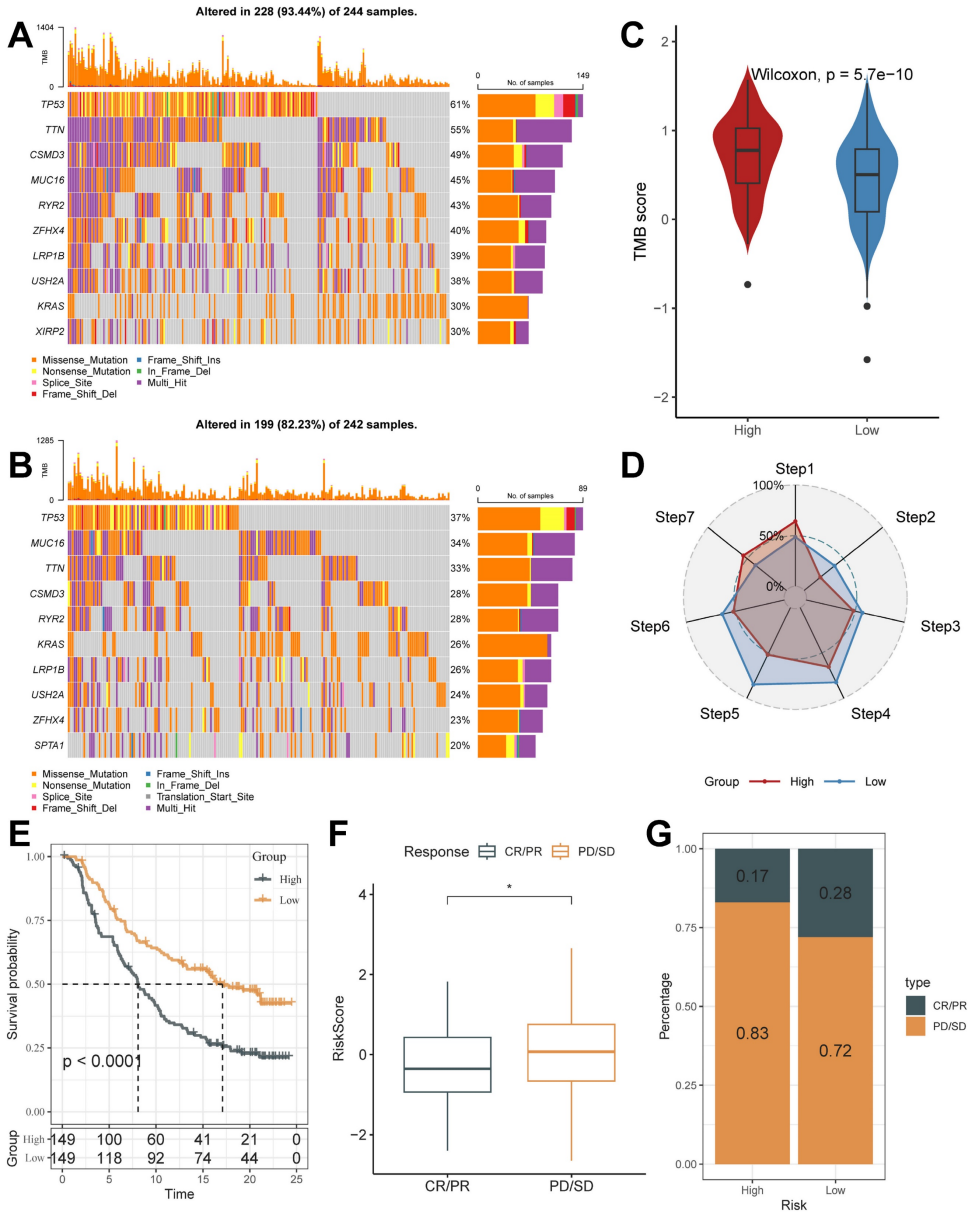
** Mutational Landscape of riskscore groups and Application of Riskscore in Immunotherapy Prediction.** (A and B) Unique gene mutation profiles of the high- and low-risk groups. (C) Different TMB Score of the high and low-risk groups (Wilcoxon test). (D) The anti-cancer immune activity of the risk groups in the cancer-immunity cycle. (E) Kaplan-Meier curves for the high and low-risk score groups in the IMvigor210 cohort. (F) The difference in morbidity risk scores between the PD/SD and CR/PR groups in the IMvigor210 cohort (Wilcoxon test). (G) Distribution of anti-PD-1 treatment responses in different risk subgroups. *, p < 0.05. **, p < 0.01. ***, p < 0.001.

The mutation profiles were distinctly varied in the top 10 genes in the high and low-risk groups (Figure 8A and 8B). Mutation rates varied widely, even in genes shared between them. For example, *TP53*, a well-recognized oncogene, was found to be mutated in 61% of the high-risk group and only 37% of the low-risk group. Previous studies have shown that *TP53* mutations are the most enriched mutations in LUAD at the invasive stage and that *TP53* is a key mediator of lung cancer invasion 49. This result suggests that the high riskscore group is more aggressive. Also, we noticed that the tumor mutation burden (TMB) was significantly higher in the high-risk group than in the low-risk score group (Figure 8C).

Cancer treatment has been revolutionized by the advent of cancer immunotherapy, the success of which relies heavily on the development and activation of immune cells in the system 50. Systematic tracking of tumor immune phenotypes is essential for understanding tumor immune mechanisms and improving the clinical efficacy of immunotherapy. Before performing the immunotherapy response prediction analysis, we first analyzed the cancer-immunity cycle among groups (Figure 8 D). The results showed that the high score group had higher expression in Step 1: Release of cancer cell antigens, and Step 7: Killing of cancer cells; while the low score group had higher expression in Step 2: Cancer antigen presentation, the Step 3: Priming and activation, Step 4: Trafficking of immune cells to tumors, Step 5: Infiltration of immune cells into tumors, and Step 6: Recognition of cancer cells by T cells. This result matches the results of mutational analysis that high TMB levels in high-risk subgroups lead to a higher potential for neoantigen production by tumors, which in turn promotes steps one and seven.

On this basis, we analyzed the response to PD-L1 blockade immunotherapy in the IMvigor210 and GSE78220 cohorts.348 patients in the IMvigor210 cohort showed different responses to anti-PD- L1 receptor blockers, including stable disease (SD), partial remission (PR), complete remission (CR), and disease progression (PD). In the IMvigor210 cohort, the high-risk group had worse survival outcomes (Figure 8E), and its proportion of PR and CR after treatment was higher (Figure 8F and 8G). It was also validated in the GSE78220 cohort (Supplementary Figure 4B-4D). We hypothesized that the low-risk subgroup with better immunotherapy gains may be associated with its active status in multiple steps of tumor immunity. In addition, we used SubMap analysis to predict immune responsiveness and showed that the low-scoring group may benefit more from PD-1 therapy (Supplementary Figure 4E). The above results suggest that TB-related Riskscore can predict immunotherapy responsiveness in different individuals.

In the above analysis, the high-risk group demonstrated a worse prognosis and fewer levels of immune cell infiltration, had invasion-associated mutational signatures (e.g., *TP53* mutations), and demonstrated low benefitability in tumor immunotherapy. In summary, the TB-related Riskscore is a good predictor, and an increase in score tends to predict the onset of a poor prognosis and low profitability in immunotherapy.

### Screening and validation of key genes

**Figure 9:**
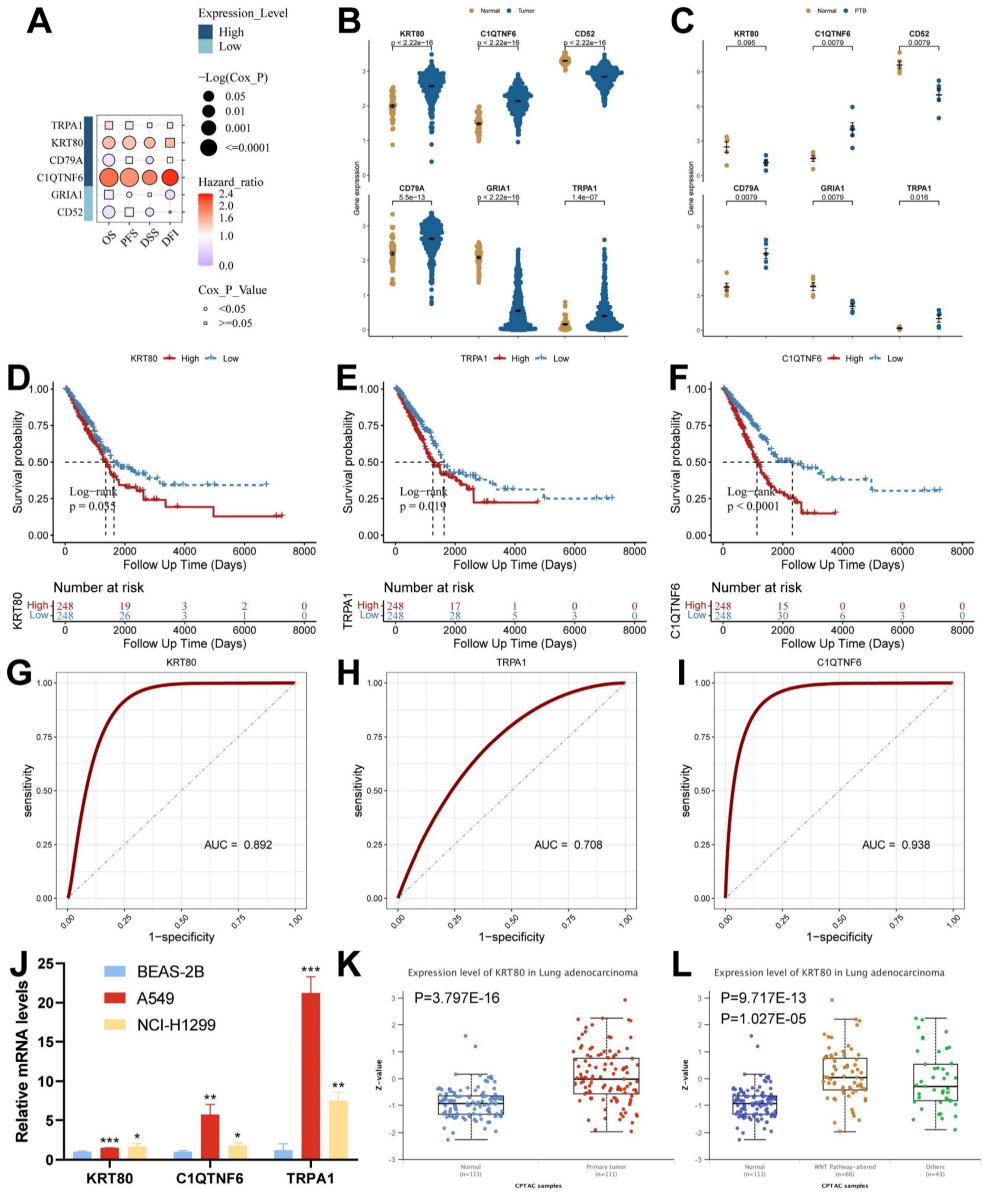
** Analysis of expression, prognosis, and diagnostic value of model genes.** (A) The Kaplan-Meier analysis of model genes in the TCGA cohort. (B) Expression of model genes in LUAD (Wilcoxon test). (C) Expression of model genes in TB (Wilcoxon test). (D-F) Survival analysis of model genes associated with overall survival in the TCGA cohort. (G-I) The Receiver Operating Characteristic Curve of KRT80, TRPA1, and C1QTNF6 in the TCGA cohort. (J) RT-qPCR assay for KRT80, TRPA1, and C1QTNF6 expression (t test). (K) Protein expression of KRT80 in lung adenocarcinoma samples from CPTAC. (L) Expression of KRT80 after alteration of the WNT pathway. *, p < 0.05. **, p < 0.01. ***, p < 0.001.

To screen the key genes, we analyzed them using the GSCA database, which showed that high expression of *KRT80* and *C1QTNF6* in cancer tissues was significantly associated with the overall survival of the samples (Figure 9A and Supplementary Table 6). Among the six model genes, *C1QTNF6*, *CD52*, *CD79A*, *GRIA1*, and *TRPA1* had similar expression trends in both diseases. In contrast, *KRT80* had opposite expression trends in TB versus LUAD (Figure 9B and 9C). We analyzed model genes in the TCGA cohort (Supplementary Figure 5A), and the results showed that differential expression of model genes can influence patient prognostic outcomes. Specifically, we found that *KRT80*, *TRPA1*, and *C1QTNF6* had worse prognostic performance when highly expressed (Figure 9D-9F), and all three genes had good diagnostic potential (Figure 9G-9I). Therefore, we concluded that *KRT80*, *TRPA1*, and *C1QTNF6* derived from TB-LUAD-related genes could be used as novel markers for LUAD. We explored gene expression in lung adenocarcinoma cell lines using RT-qPCR. The results showed that compared to BEAS-2B cells, *KRT80*, *TRPA1*, and *C1QTNF6* were highly expressed in two lung adenocarcinoma cell lines (A549 and NCI-H1299) (Figure 9J). In addition, *KRT80* protein expression was found to be significantly higher in lung adenocarcinoma samples than in normal samples in the UALCAN database (Figure 9K), while the level of *KRT80* protein expression was elevated after alteration of Wnt pathway activity (Figure 9L).

**Figure 10:**
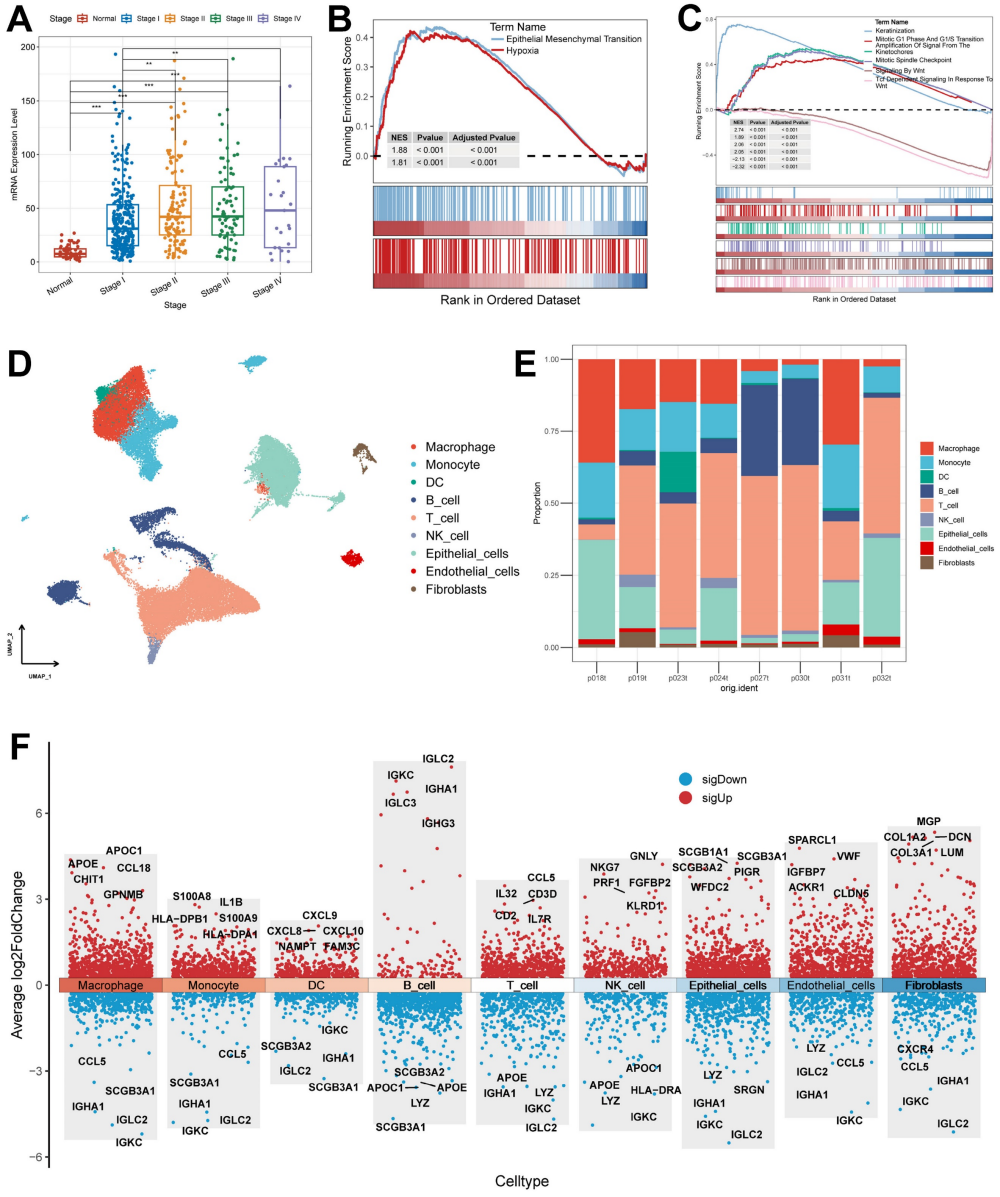
** Association of KRT80 with clinical features and pathways and explanation of cellular subpopulations.** (A) Expression of KRT80 in different Stages (Wilcoxon test). (B) Hallmark pathway enrichment results of KRT80 high expression group. (C) Reactome pathway enrichment results of KRT80 high expression group. (D) UMAP plots are used for descending clustering sorting. (E) The proportion of 9 cell types in 8 different samples. (F) Nine cell types for differential gene expression demonstration. *, p < 0.05. **, p < 0.01. ***, p < 0.001.

By analyzing the relationship between clinical features and *KRT80* expression, we found that *KRT80* expression varied in different pathological stages, and KRT80 expression increased with increasing stage, which may predict that *KRT80* is associated with tumor metastatic progression (Figure 10A).

This is consistent with the altered protein expression of *KRT80* in response to altered Wnt pathway activity, i.e., *KRT80* is associated with tumor metastasis and may act through the Wnt pathway. To further explore its relationship with LUAD progression, we categorized the TCGA cohort into high and low-expression groups based on *KRT80* expression. In the Hallmark gene set, the high expression group was enriched to epithelial-mesenchymal transition and hypoxia pathway (Figure 10B). To explore more detailed biochemical alterations, we obtained the Reactome gene set and performed enrichment analysis, which showed that epithelial-mesenchymal transition, Wnt pathway, and tumor cell division-related pathway were highly active in the *KRT80* high expression group (Figure 10C). Considering the high diagnostic value of *KRT80* and *C1QTNF6*, we further analyzed them.

First, we acquired and processed single-cell data from eight lung adenocarcinoma samples. After visualization using the UMAP algorithm, we classified the cell clusters into nine cell types using Marker genes (Figure 10D) and evaluated the distribution ratio of these nine cell types in the eight samples (Figure 10E). The results of the evaluation of different cell differential genes similarly illustrate the accuracy of the analysis and annotation (Figure 10F).

**Figure 11:**
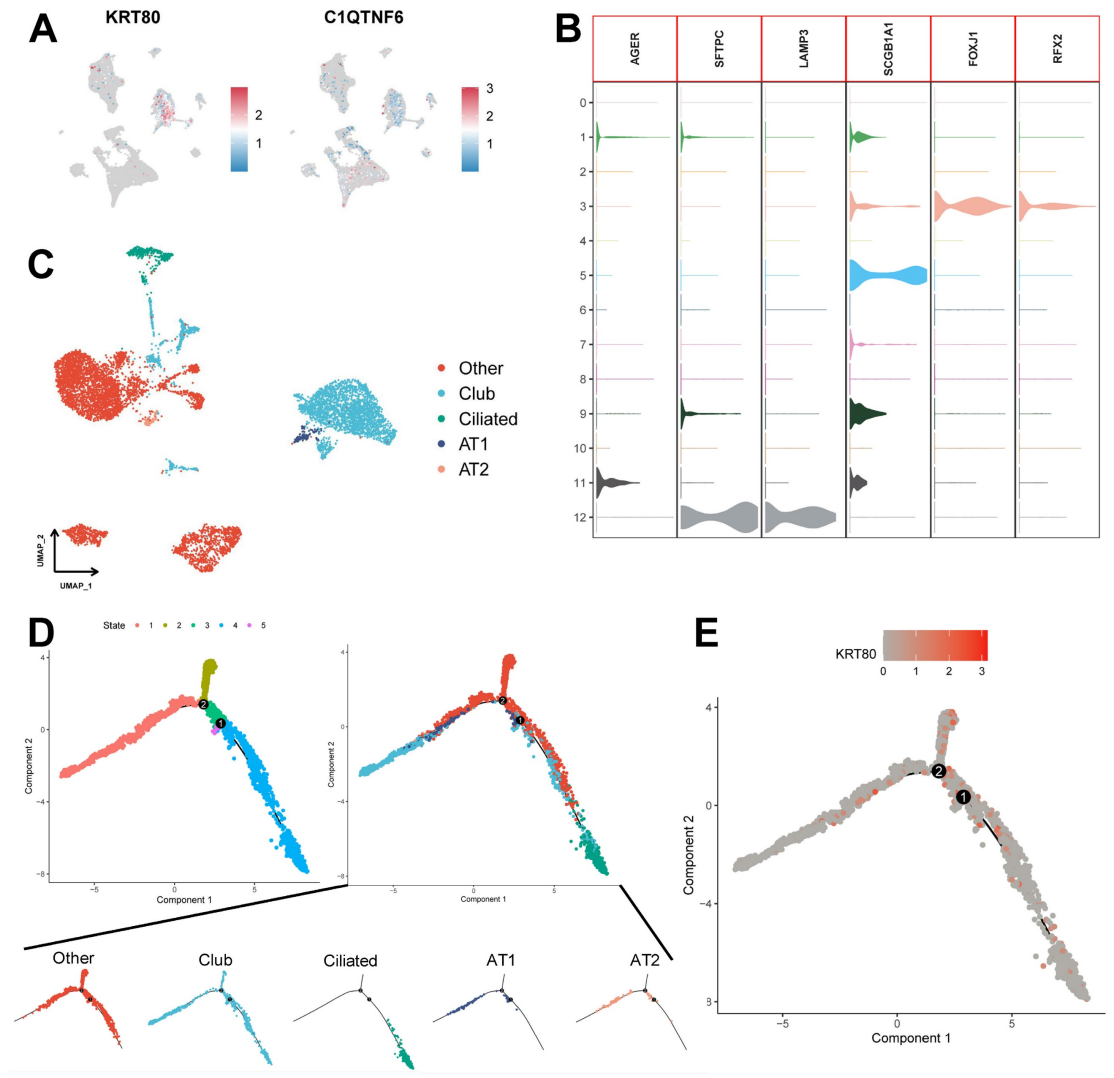
** Potential role of KRT80 in epithelial cell differentiation.** (A) Expression of KRT80 and C1QTNF6 in cell clusters. (B) Expression levels of epithelial cell-associated Marker genes. (C) UMAP plots demonstrate the results of epithelial cell annotations. (D) Demonstration of meticulous epithelial cell differentiation trajectories. (E) Expression of KRT80 in differentiation trajectories.

We assessed the expression of six model genes in cell clusters (Supplementary Figure 5B), and the results showed that *KRT80* and *C1QTNF6* had high expression in specific cell types (Figure 11A). In particular, *KRT80* was highly expressed in epithelial cells. Therefore, we extracted epithelial cells separately for annotation. Normal epithelial cells are mainly composed of four different subpopulations, including alveolar type I (AT1) and type II (AT2), Club cells, and ciliated cells, which express clear epithelial markers (Figure 11B and Supplementary Table 7). The results of the UMAP algorithm showed that epithelial cells subdivided into different types had a clear distinguishability (Figure 11C), in which AT1 and AT2 could initiate LUAD in the distal airway 51. For types that could not be identified, we defined them as the Other types, which we presumed to be malignant cells. Using data after epithelial cell annotation, we constructed cell differentiation trajectories (Figure 11D and Supplementary Figure 5C) to probe the gene expression programs associated with tumor progression. Indeed, the transcriptional states in the trajectories revealed the normal differentiation pathway of the tumor as well as progression-related changes.

First, Ciliated cells and alveolar cells were located at different positions in the differentiation trajectory, which suggested their different differentiation states. Meanwhile, both of these cells are in the Club cell differentiation track, which indicates that Club cells are in an intermediate differentiation state 52. In addition, the Other type of cells had few cells at the beginning of the differentiation process but were present in most of the subsequent differentiation trajectories and formed distinctive branching structures. This may suggest a malignant progression of epithelial cells as the differentiation program becomes dysregulated. We further explored the expression of *KRT80* (Figure 11E) in the differentiation trajectory and found that it was predominantly located in the Other type, which may suggest that *KRT80* is involved in the malignant progression of lung adenocarcinoma tumors.

## Discussion

Tuberculosis and lung adenocarcinoma have been linked in clinical practice 53; for example, lung tumors and oncology therapeutic agents are associated with immunosuppression 54, 55, which often leads to Mycobacterium infection, further inducing tuberculosis. Chronic inflammation of tuberculosis is also carcinogenic 56, 57. However, the role and potential association of TB in lung adenocarcinoma remains unclear. In the present study, we compared the molecular signatures of TB and LUAD to discover common pathogenic molecules, TB-LUAD-shared genes. Consensus clustering of the TCGA cohort based on TB signature genes characterized three classes of molecular subtypes with distinctive clinical presentations, immune features, cellular infiltration, and pathway alterations. This result indicates that TB has a unique impact in the progression of lung adenocarcinoma. Meanwhile, we developed and validated a TB-related Riskscore with good clinical predictive efficacy. In this study, for the first time, a comprehensive bioinformatics analysis was utilized to reveal common biological processes and similar transcriptional changes between lung adenocarcinoma and tuberculosis.

Tuberculosis has been a serious threat to human life and health, and its cause is due to Mycobacterium tuberculosis infection. Lung adenocarcinoma, as one of the most common lung cancers, requires further development of its diagnostic and therapeutic features. Lung cancers often exhibit adherent hyperplasia with the presence of cancer-associated fibroblasts (CAFs), Extracellular Matrixes (ECMs), and immune cells. Among them, ECMs play an important role in regulating tissue inflammation and promoting tumor metastasis 58; at the same time, signaling factors in TME remodel ECMs, which in turn lead to cellular transformation promoting cancer growth and distant metastasis 59, 60. Chai et al. showed that lung stromal remodeling and fibrillogenic collagen deposition are common pathogenic features TB and LUAD share 26. Considering the relationship between EMCs and TME and the presence of common features of TB and LUAD in ECMs, then the potential mechanisms of the two disorders in the landscape of cellular infiltration deserve to be further explored. In addition, Mycobacterium tuberculosis produces malignant transforming precursors (including genomic instability and mutations, inflammation), and the development of tuberculosis further contributes to the development of lung cancers 16, more prominently lung adenocarcinoma, which in turn is a risk factor for the pathogenesis of tuberculosis 61. In summary, multiple lines of evidence suggest that the two share similar characteristics in their development and cellular infiltration landscapes.

Based on TB and LUAD lung transcriptional profiling data, we identified 380 common differentially expressed causative genes in both diseases and defined them as TB-LUAD-shared DEGs. The enrichment analyses of these genes indicate that they may be key LUAD-associated disease-causing mediators affected by TB infections. We screened for genes associated with overall survival in LUAD patients in TB-LUAD-shared DEGs and further defined them as TB Signature genes, which may play a role in TB influencing the progression of LUAD. Thus, we identified three molecular subtypes with unique expressions based on TB Signature genes using Consensus Clustering. TB Signature genes showed different expression profiles among different subtypes and similar expression profiles to the differential expression profiles of LUAD and TB samples, with C1 subtypes showing similar gene expression to TB samples, C2 and C3 subtypes showing similar gene expression to LUAD samples. This result indicates that LUAD has different progression and prognostic outcomes under the influence of TB pathogenic factors. The results of Kaplan-Meier analysis showed significant prognostic differences among the three subtypes, i.e., subtype C1 had a favorable prognosis, while subtypes C2 and C3 had a poorer prognosis. In order to depict the three subtypes with differential prognostic outcomes, we explored them in terms of clinical features, cellular infiltration, and pathway change. And finally, the three subtypes were found to be highly compatible with the immunophenotyping features of Immune inflamed, Immune exclued, and Immune desert. We hypothesized that the three classes of LUAD molecular subtypes derived from the tuberculosis transcriptome could be distinguished into three phenotypes with distinctive features. The C1 subtype, Immune inflamed, is characterized by INF-γ signaling expression, B-cell infiltration, as well as massive immune cell infiltration and inflammatory response; the C2 subtype, Immune desert, is characterized by tumor cell proliferation, glycolysis, WNT Signaling expression, and immunosuppression; C3 subtype is Immune exclude, characterized by stromal activation, angiogenesis, and immune cell infiltration and rejection. This result likewise suggests the possibility that LUAD has a number of different disease progression possibilities in the presence of TB causative factors.

We further mined genes that are prognostically relevant in LUAD by comparing TB Signature genes with DEGs from the TCGA cohort and obtaining intersections. Based on this, we constructed and validated a Riskscore scheme based on TB signature in six LUAD cohorts and characterized two subgroups with significant differences. Compared to the low-risk group, the high-risk group had a worse prognosis and a higher proportion of C1 subtypes, which also had a worse performance in T Stage, N Stage, M Stage, and Stage. Meanwhile, the C2 subtype showed high Riskscore, and the C1 subtype showed low Riskscore. In addition, with the increase of TB-related Riskscore, worse outcome was shown in T Stage and Stage. We explained the different prognostic outcomes between subtypes in terms of immune cell infiltration, which was significantly higher with decreasing Riskscore, accompanied by the expression of more antigen presentation-related transcripts. Similar immune results were also obtained in the cancer-immunity cycle. Subgroups differed significantly at the mutation level, with the high-risk group having higher tumor mutation loads and mutation levels in genes such as *TP53*. Whereas *TP53* mutations were associated with aggressive lung adenocarcinomas, and high *TP53* mutations tended to herald the initiation of invasion 62.

Further, we explored the predictive efficacy of TB-related Signature in immunotherapy. Consistent results were obtained when applying our model to the IMvigor210 and GSE78220 cohorts, i.e., low-scoring patients achieved better clinical benefit after anti-PD-L1 and anti-PD-1 therapy. Meanwhile, the low-risk group in the Submap algorithm showed more benefit in PD-1 therapy. These results suggest that TB-related Riskscore can be a valid predictor of immunotherapy response. We speculate that the low-risk subgroup with better immunotherapy benefits may be associated with its active status in multiple steps of tumor immunity.

The six model genes in the TB-related Riskscore scheme serve as good predictors in LUAD and have been demonstrated in a variety of cancers. For example, Keratin 80 (*KRT80*) is a human IF type II epithelial keratin gene, which is involved in the formation of IF heterodimers in a variety of epithelial cells. It is highly expressed in esophageal, gastric, colorectal, and breast cancers and is associated with tumor proliferation and metastasis 63-66. Li et al. have shown that it promotes colorectal cancer migration and invasion through the agonism of the AKT pathway 67. C1q And TNF Related 6 (*C1QTNF6*) is a protein-coding gene that has been shown to play a role in many types of cancers, contributing to the proliferation of cancer cells in gastric cancer 68 and inhibiting apoptosis in oral squamous cell carcinoma 69. We verified the expression of *KRT80*, *C1QTNF6*, and *TRPA1* in LUAD by RT-qPCR experiments. For *KRT80*, which was differentially expressed in TB and lung adenocarcinoma, we found that with its elevated expression often predicted the occurrence of poor clinical staging. Also, enrichment analysis showed that *KRT80* was associated with the epithelial-mesenchymal transition. At the single-cell transcriptome level, we found that *KRT80* has a role in epithelial cell differentiation and is associated with the malignant progression of LUAD. *KRT80* expression levels in the LUAD cohort were correlated with clinicopathologic features of the patients, and the study further revealed the impact of *KRT80* on the disease progression and clinical indices of LUAD patients. The potential marker *KRT80* may play an important role in the cancer cell function and the prognosis of cancer patients.

Inevitably, this study needs to account for some limitations. First, the study cohorts were drawn from public databases, which results in an inherent case selection bias that may affect the results, and more convincing prospective studies are needed to confirm our findings. Second, due to the limited sample size, large-scale cohort studies are essential to assess the value of the model. Meanwhile, based on the bioinformatics identification of TB-related Riskscore as well as its model genes, they still require further experiments to investigate their underlying biological mechanisms.

## Conclusion

Taken together, we identified a class of key LUAD pathogenic mediators affected by TB infection through molecular characterization of TB and LUAD, and identified three distinct molecular subtypes. We established and validated a TB-related Riskscore that accurately predicts patient survival outcomes and has good predictive efficacy for immunotherapy. In addition, model genes were validated using RT-qPCR experiments and single-cell analysis. In conclusion, this study reveals a possible pathogenic association of TB in the progression of lung adenocarcinoma. We hope that the results of this study will help to advance the research related to the potential link between lung adenocarcinoma and TB, pathogenesis, and therapeutic targets.

## Supplementary Material

Supplementary figures and table.
