# Supplementary material for: Tuberculosis to lung cancer: application of tuberculosis signatures in identification of lung adenocarcinoma subtypes and marker screening
Source: J Cancer. 2024 Aug 13;15(16):5329–50. doi: 10.7150/jca.97898 (PMC11375533; doi:10.7150/jca.97898)
Supplement: Supplementary file 1 — Supplementary figures and table. [file jcav15p5329s1.pdf]

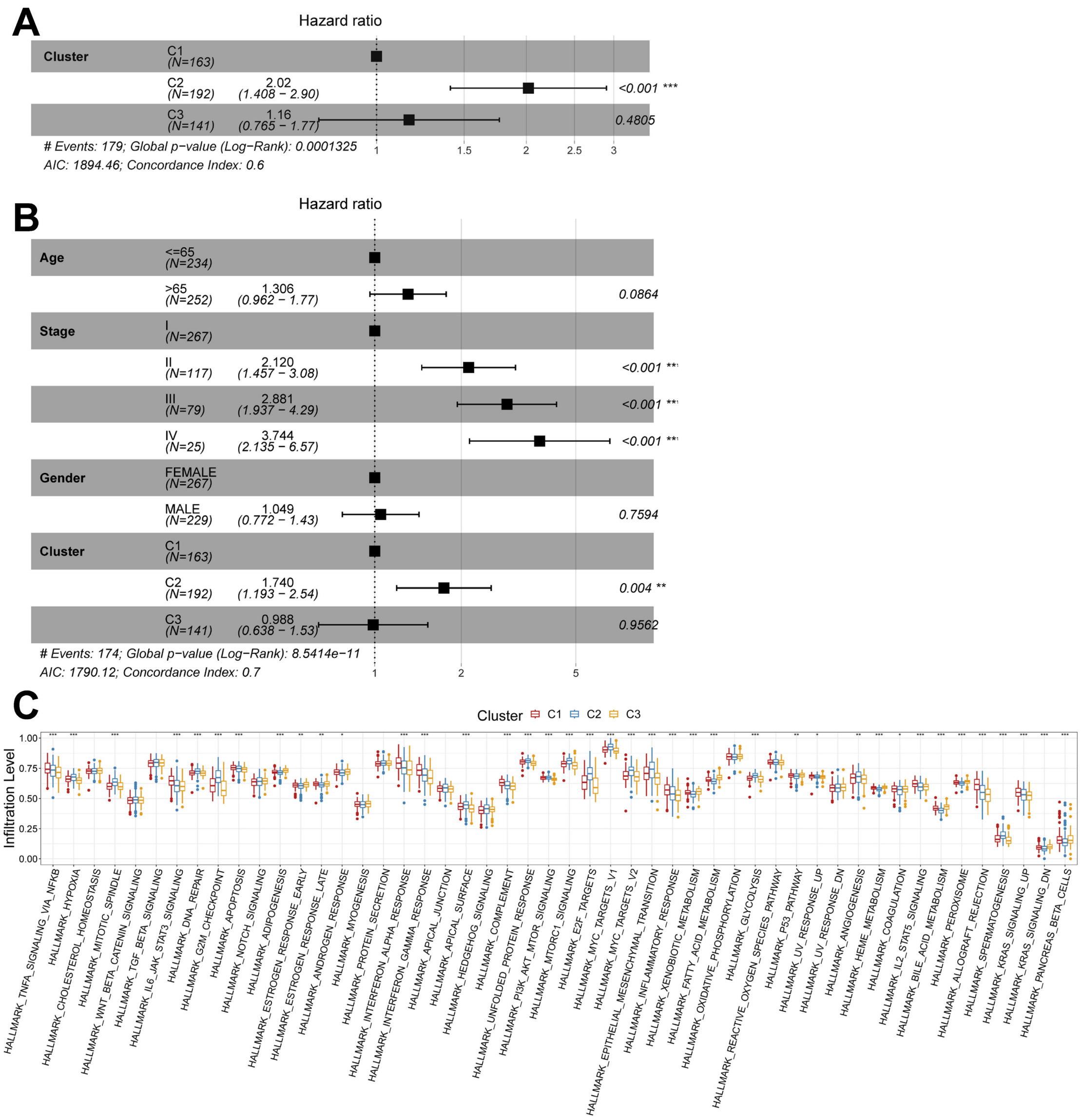

Supplementary Figure 1. Clinical and molecular characterization of subtypes

(A) Forest plot of the univariate Cox model demonstrating the relationship between Cluster subtype and overall survival. (B) Forest plot representation of the multivariate Cox model depicting the relationship between Cluster subtype and overall survival (OS) adjusted for age, staging, and age. (C) Box plots demonstrating the enrichment level of the Hallmark pathway between subtypes (Kruskal test). *, $p < 0.05$. **, $p < 0.01$. ***, $p < 0.001$.

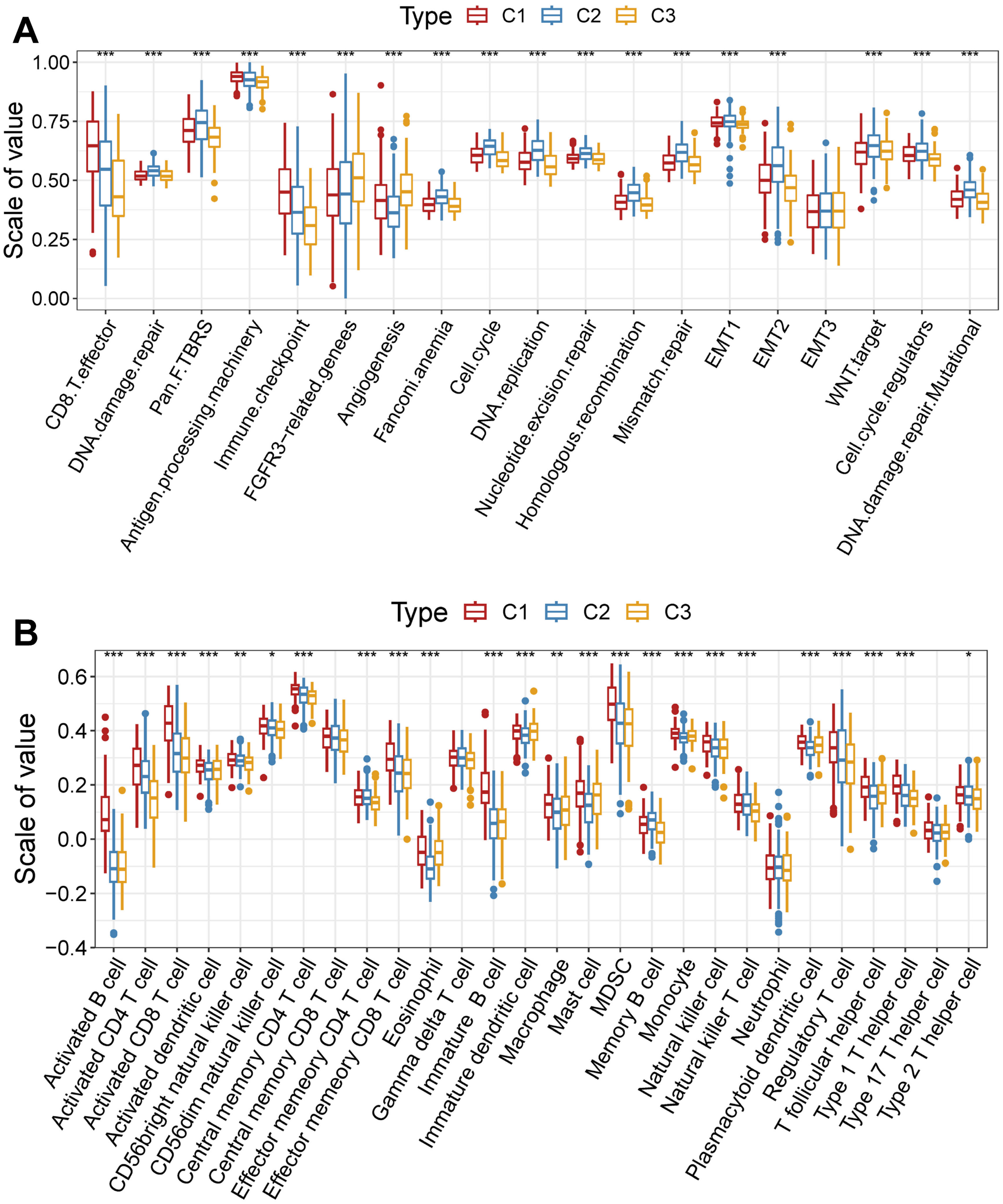

Supplementary Figure 2. Enrichment analysis results and cellular infiltration of subtypes

(A) Box plot showing the differential enrichment results of molecular subtypes in the Mariathasan gene set (Kruskal test). (B) Cellular infiltration levels between subtypes were calculated using ssGSEA (Kruskal test). *, $p < 0.05$. **, $p < 0.01$. ***, $p < 0.001$.

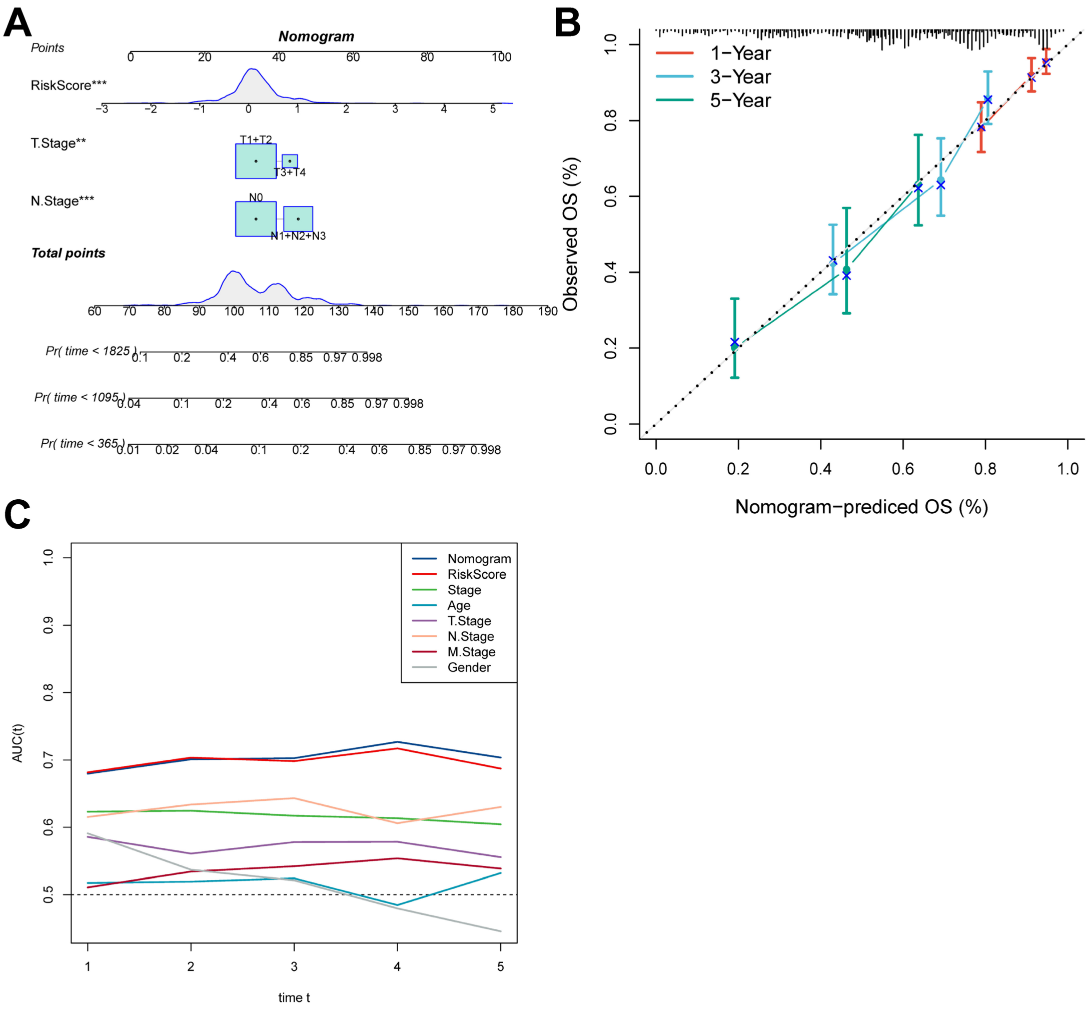

Supplementary Figure 3. Constructing predictive nomograms for TB-Related Riskscore

(A)Nomogram of Riskscore versus clinical features demonstrating and predicting the probability of survival. (B)Nomogram calibration curves for 1, 3, and 5 years. (C) Assessment of the predictive power of the characteristics.

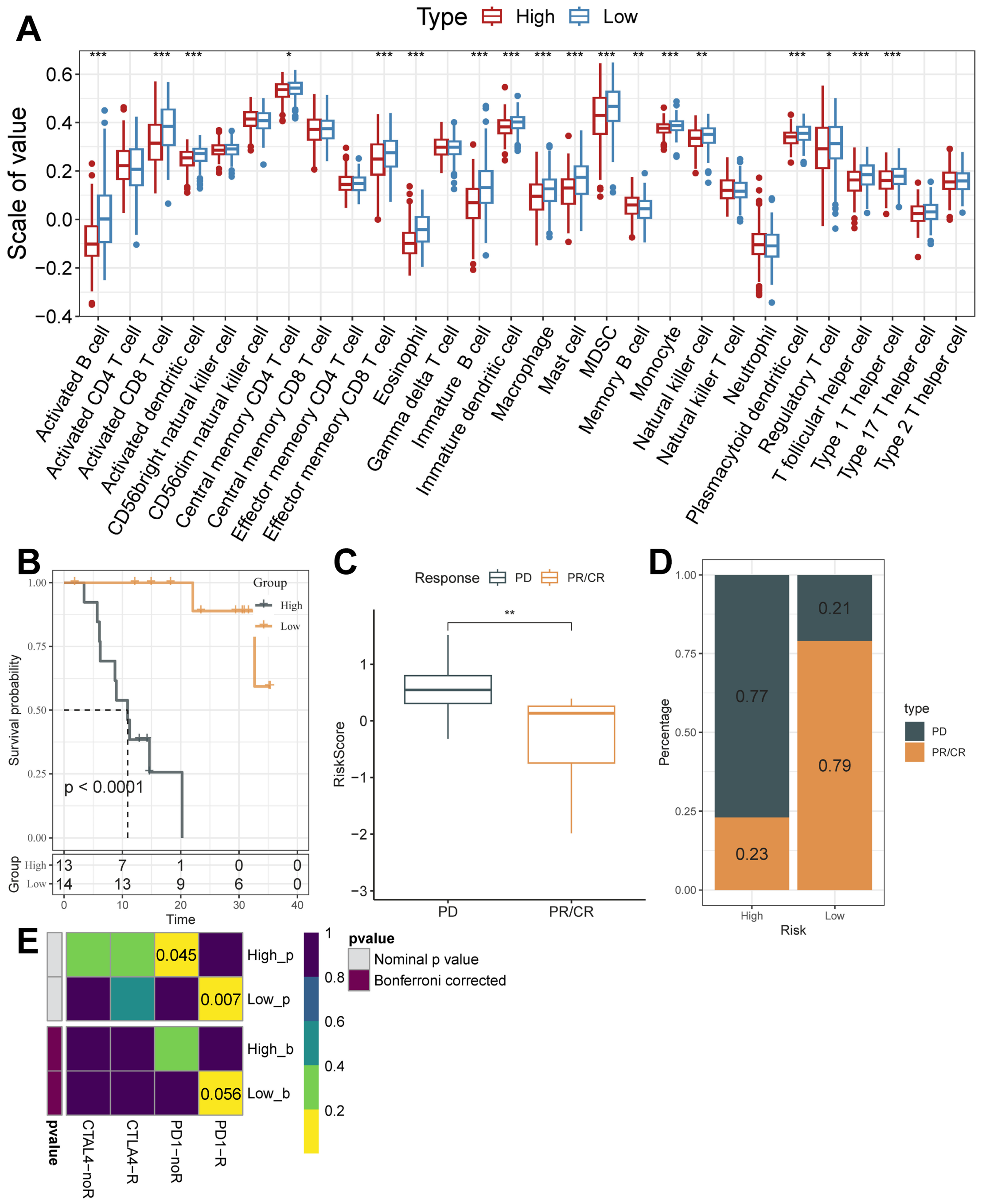

Supplementary Figure 4. Cellular infiltration and immunopredictive efficacy of TB-related Riskscore

(A) Box line plot showing ssGSEA results for high and low-risk groups. (B)Kaplan-Meier curves for the high and low-risk score groups in the GSE78220 cohort. (C)Difference in morbidity risk scores between the PD/SD and CR/PR groups in the GSE78220 cohort (Wilcoxon test). (D)Distribution of anti-PD-1 treatment responses in different risk subgroups. (E) Heatmap demonstrating the results of the Submap algorithm for the prediction of immunization efficacy. *, $p < 0.05$. **, $p < 0.01$. ***, $p < 0.001$.

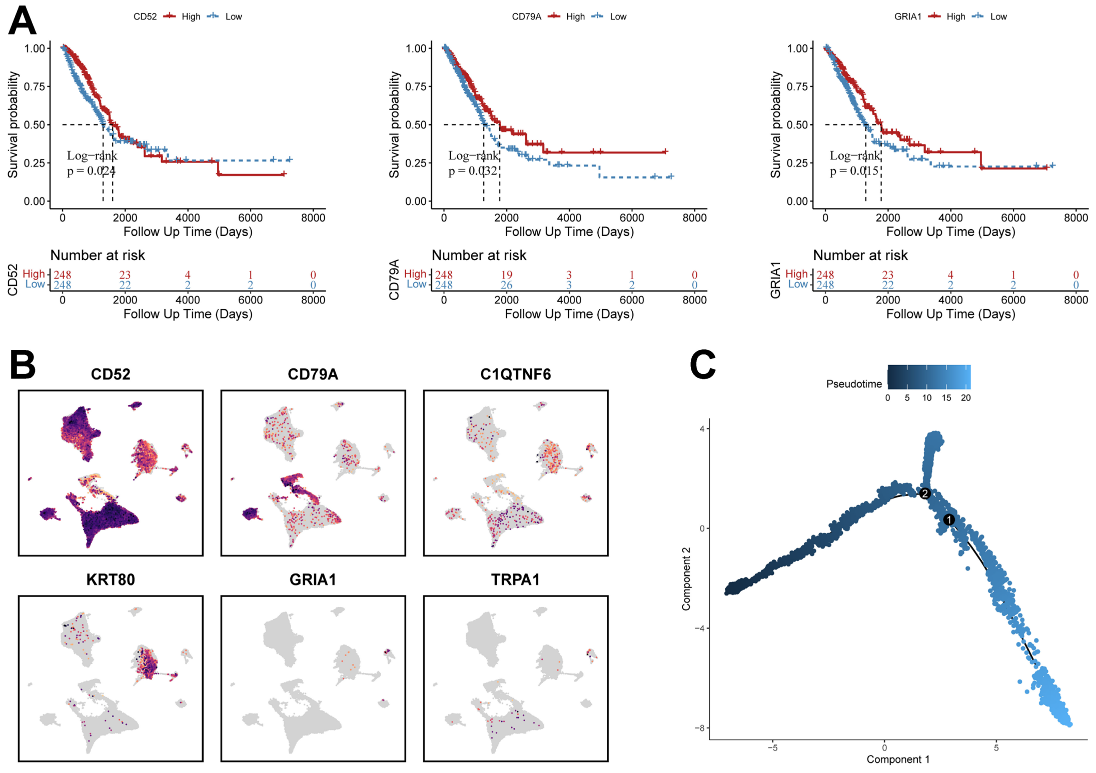

Supplementary Figure 5. Survival analysis and single-cell analysis of model genes

(A) Kaplan-Meier curves of high and low expression groups of CD52, CD79A, GRIA1. (B) Expression of model genes in cell clusters. (C) Visualization of Pseudotime results.

Supplementary Table 1. The sequences of primers used in real-time PCR.

Gene Symbol	Sequence (5'-3')	
KRT80	Forward primer	CAG CTG AAG AAG GAC CTG GA
	Reverse primer	GAC ACA TCC TTC ACC TGT GC
C1QTNF6	Forward primer	GAA AGG GTC TTT GTG AAC CTT GA
	Reverse primer	CTG CGC GTA CAG GAT GAC AG
TRPA1	Forward primer	TAG ACC CAG TGG AGA AGG GA
	Reverse primer	AGC TCT GTG AAG CAT GGT CT
